# Actions at a glance: The time course of action, object, and scene recognition in a free recall paradigm

**DOI:** 10.3758/s13415-025-01272-6

**Published:** 2025-02-26

**Authors:** Maximilian Reger, Oleg Vrabie, Gregor Volberg, Angelika Lingnau

**Affiliations:** Faculty of Human Sciences, University of Regensburg, Universitätsstraße 31, 93053 Regensburg, Germany

**Keywords:** Action recognition, Action understanding, Free recall, Natural scene, Object recognition, Perception, Scene recognition

## Abstract

Being able to quickly recognize other people’s actions lies at the heart of our ability to efficiently interact with our environment. Action recognition has been suggested to rely on the analysis and integration of information from different perceptual subsystems, e.g., for the processing of objects and scenes. However, stimulus presentation times that are required to extract information about actions, objects, and scenes to our knowledge have not yet been directly compared. To address this gap in the literature, we compared the recognition thresholds for actions, objects, and scenes. First, 30 participants were presented with grayscale images depicting different actions at variable presentation times (33–500 ms) and provided written descriptions of each image. Next, ten naïve raters evaluated these descriptions with respect to the presence and accuracy of information related to actions, objects, scenes, and sensory information. Comparing thresholds across presentation times, we found that recognizing actions required shorter presentation times (from 60 ms onwards) than objects (68 ms) and scenes (84 ms). More specific actions required presentation times of approximately 100 ms. Moreover, thresholds were modulated by action category, with the lowest thresholds for locomotion and the highest thresholds for food-related actions. Together, our data suggest that perceptual evidence for actions, objects, and scenes is gathered in parallel when these are presented in the same scene but accumulates faster for actions that reflect static body posture recognition than for objects and scenes.

**Supplementary information:**

The online version contains supplementary material available at 10.3758/s13415-025-01272-6.

## Introduction

Being able to recognize goal-directed actions is crucial for our ability to successfully interact with the world around us. To accomplish this task, information originating from a variety of different sources regarding body posture, movement kinematics, the scene in which the action takes place, and objects involved in the action needs to be analyzed and integrated (Bach et al., 2014; Lingnau & Downing, 2024; Wurm et al., 2012). The importance of these individual sources of information for recognizing actions likely varies across different types of actions (Kabulska & Lingnau, 2022). For instance, object information may be particularly crucial for tool-related actions but less so for communicative actions like “waving” or “shaking hands.” Additionally, the role of these information sources likely depends on the observer’s task, such as recognizing the type of sport another person is engaged in versus recognizing whether a player committed a foul against another player. While several of these components have been shown to contribute to action recognition, the time that is required to accumulate perceptual evidence for these components, and the temporal order in which they are being recognized is not well understood. However, such knowledge is crucial for building biologically plausible models of action recognition.

Several behavioral studies using static images as stimulus material revealed that humans can distinguish between different types of actions with presentation times as short as several tens of milliseconds (Zhuang & Lingnau, 2022; Fei-Fei et al., 2007). With comparable presentation times, human participants can recognize body postures (Glanemann et al., 2016) and event role information (i.e., who acted on whom; Hafri et al., 2018). Together, these findings suggest that actions and their components can be recognized with relatively short stimulus presentation times (see also Hafri & Firestone, 2021, for a related discussion). By contrast, Dobel et al. (2007) obtained that participants were able to correctly identify which of two actions was present in a line drawing of a complex scene in only 19% of all trials when they were presented for 100 ms (followed by a mask), with performance still relatively low (46%) even when they were presented for 300 ms. Thus, estimates regarding the precise presentation time that is required to accumulate enough information to enable successful action recognition vary substantially across studies.

On an implementational level, using dynamic stimuli, several EEG and MEG studies showed that it is possible to distinguish between different types of observed actions based on activation patterns as early as 200 to 250 ms after stimulus presentation (Dima et al., 2022; Isik et al., 2018; Tucciarelli et al., 2015). Additionally, using multivariate analyses of EEG data, Dima et al. (2022) revealed a temporal gradient underlying the processing of actions depicted in videos, with visual features and features related to objects and scenes being processed before action-related features.

Both the scene and the objects involved in the action are assumed to contribute to action recognition (Bach et al., 2014; Wurm et al., 2012, 2017; Wurm & Schubotz, 2012, 2017). Scene information has been shown to be extracted rapidly from static images (Fei-Fei et al., 2007; Potter, 1975), with perceptual thresholds for global scene properties around presentation times as short as 30–40 ms, and for basic scene category information around 30–70 ms (Greene & Oliva, 2009). Moreover, using static images, scene information has been shown to have an impact on object recognition, and vice versa (Biederman, 1972; Joubert et al., 2007; Krugliak et al., 2023; Wiesmann & Võ, 2023). For example, objects can be identified faster and more accurately when they are embedded in semantically congruent compared to incongruent scenes (Biederman et al., 1982; Davenport & Potter, 2004). Bar (2004) suggested that rapidly extracted low spatial frequency information provides vague shape information and simultaneously activates context frames that provide information about likely objects in a specific scene. According to this view, both sources of information are combined, resulting in an expectation of the most likely object given the current shape information in a specific scene, which in turn facilitates object recognition. Similar mechanisms might be involved in the recognition of actions (see also Bach et al., 2014; Lingnau & Downing, 2024). In line with this view, like object recognition, action recognition has been shown to profit from congruent scene information in dynamic stimuli, such as cooking in a kitchen compared to an office (Wurm & Schubotz, 2012, 2017), even in young children (Wurm et al., 2017). Given that scene recognition has been shown to be relatively fast (Fei-Fei et al., 2007; Greene & Oliva, 2009; Potter, 1975), it has been proposed that rapidly extracted scene information leads to preactivation of likely actions (Wurm et al., 2017), which implies that scene information is available earlier than information regarding the action. However, to our knowledge, the stimulus presentation time that is required to extract scene and action information has not been directly compared using the same analytical approach. It remains unclear whether scene-related information is available earlier than action-related information or vice versa.

Using a free-recall paradigm, Fei-Fei et al. (2007) found that with stimulus presentation times as short as 107 ms, scene information as well as object information can be recognized. Moreover, object and scene recognition performance was significantly correlated at low presentation times, which the authors interpret to indicate that either one helps the other, and/ or that both processes share resources (Fei-Fei et al., 2007).

Based on these previous studies, we aimed (1) to determine the perceptual threshold for action recognition, and (2) to compare thresholds for the recognition of actions, objects (regardless of whether they were part of the action), and scenes. Moreover, we reasoned that it is plausible that evolutionary relevant actions such as “attacking” or “eating” might be perceived faster than other actions (Lingnau & Downing, 2024; Wurm et al., 2017). We thus aimed (3) to test whether perceptual thresholds for action recognition are modulated by the category to which the action belongs (e.g., locomotion, communication, or food-related actions; Kabulska & Lingnau, 2022; Tucciarelli et al., 2019).

To address these points, we conducted a free recall experiment (Fei-Fei et al., 2007). We hypothesized that scene information can be accumulated faster than action and object information and that perceptual thresholds for the recognition of actions differ between categories.

## Methods

### Preregistration

The hypotheses, methods, and analyses plan of all experiments, including the pilot study were preregistered (10.17605/OSF.IO/6UTYN) on 2022-09-15, prior to data collection, which began on 2022-09-22. There was one major and several minor deviations from the preregistration; all deviations from this preregistration are listed in Supplementary Material 1 (Table S1). Additionally, for completion, we report the results of all preregistered analyses that were not reported in the main manuscript in Supplementary Material 2.

The main experiment included two stages following Fei-Fei et al. (2007). In stage 1, participants were asked to provide detailed verbal descriptions for images presented at varying presentation times. In stage 2*,* a separate group of participants rated the accuracy of these descriptions with respect to the action, object, scene and sensory information. Experimental procedures were approved by the local ethics committee at the University of Regensburg.

### Stage 1: Free recall experiment

#### Participants

Thirty (3 males, 27 females) healthy German-native speakers (age range 18–27 years; *M* = 20.5; *SD* = 2.45) with normal or corrected-to-normal vision took part in stage 1. They were recruited via social media and in a General Psychology lecture for first-year students. Most participants (N = 26) studied psychology at the time of the experiment. All participants gave informed consent to take part in the study.

#### Materials

**Fig. 1:**
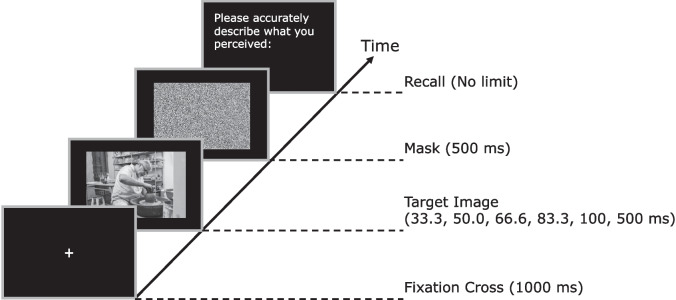
Example trial used in Experiment 1. First, a fixation cross was presented for 1000 ms. Next, the target image was presented for 33.3, 50, 66.6, 83.3, 100, or 500 ms, followed by a mask (500 ms). At the end of each trial, the participant was asked to use the keyboard to provide a detailed description of what they just saw. There was no time limit to provide this description

We used 70 images of size 600 x 400 pixels (corresponding to 8° x 6° visual angle), depicting actions in naturalistic scenes (see Fig. 1 for an example). All images were photographs and are available on OSF.^1^ We included actions belonging to the categories “Cleaning”; “Communication”; “Food”; “Leisure”; and “Locomotion,” with 14 different basic-level actions per category, selected based on a multi-arrangement experiment (see Supplementary Material 3). Masks consisted of shuffled and randomly rotated 2 x 2 pixel tiles obtained from each image (see Fig. 1 for an example).

The experiment was programmed in MATLAB, R2019a (The MathWorks Inc., 2019) using the Psychophysics Toolbox, Version 3 (Brainard, 1997) in combination with “A simple framework” (ASF v0.56) (Schwarzbach, 2011).

#### Design

We used a 6x5 within-subject-design, with the factors *presentation time* (33, 50, 67, 83, 100, and 500 ms) and *action category* (5 levels). We chose these presentation times (PT) based on Fei-Fei et al. (2007), who used similar PTs investigating the time course of object and scene recognition in a similar paradigm and based on a recent study that examined action recognition as a function of presentation time (Zhuang & Lingnau, 2022). Presentation times were balanced across images, with each image being presented five times for each PT. Presentation times were randomly permuted across images for each participant.

Each action category consisted of 14 basic-level actions (depicted as a static image), with each of the 70 different images presented once to each participant. The order of images was randomized within participants, while preventing any occurrence of two or more consecutive images of the same category.

#### Procedure

This experiment was conducted in a laboratory at the University of Regensburg. Participants received written instructions. Next, participants ran through a practice version consisting of five trials (with presentation times varying between 66.6 and 200 ms) to make sure that they understood the task. Images shown in the instructions and the practice version were not used in the main experiment.

During the experiment, participants were seated at a distance of 106 cm in front of a DELL P2219H monitor (screen resolution: 1920 x 1080 pixels, 21.5 inch), and their head position was fixed by a chin rest.

As shown in Fig. 1, each trial started with a fixation cross on a black background (1000 ms), followed by a target picture (33.3, 50, 66.6, 83.3, 100, or 500 ms) and a mask (500 ms). At the end of each trial, participants were asked to provide a detailed written description of everything they perceived and to take as much time as they needed. Pressing the Enter-Key started the next trial. Participants were not biased towards describing any specific image features, such as the action, the objects, or the background scene depicted in the image. A translated version of the instruction can be found in Supplementary Material 4. To motivate participants, an ice cream voucher was promised for the participant who gave the most detailed descriptions.

The experiment consisted of 70 trials and was conducted in one session with a 1-min break after every sixth trial. At the end of the experiment, participants were asked to fill out a demographics questionnaire and to indicate their level of exhaustion, their use of any specific strategies, and their ideas regarding the aim of the experiment. Participants were compensated with course credits and sweets. The overall procedure took approximately 3 h.

### Stage 2: Evaluation of stimulus descriptions

#### Participants

A separate group of ten healthy normal or corrected-to-normal sighted naïve participants (1 male, 9 females; age range 19–25, *M* = 20.7 years; *SD* = 2.21) took part in stage 2. All participants were students with a background in psychology. All participants signed informed consent.

#### Materials

Stage 2 was conducted by using the online platform lab.js (Henninger et al., 2022). All participants used their own laptops to run the experiment at home (except for the first session; see details below). The descriptions obtained in stage 1, and the corresponding images were used as stimuli in stage 2. Data analysis was performed in Python 3 and RStudio (Posit team, 2024; R Core Team, 2024).

#### Design

Each participant rated all 2100 descriptions obtained in stage one. Descriptions were shuffled and randomly divided into ten equally sized batches containing 210 descriptions each.

#### Procedure

**Fig. 2:**
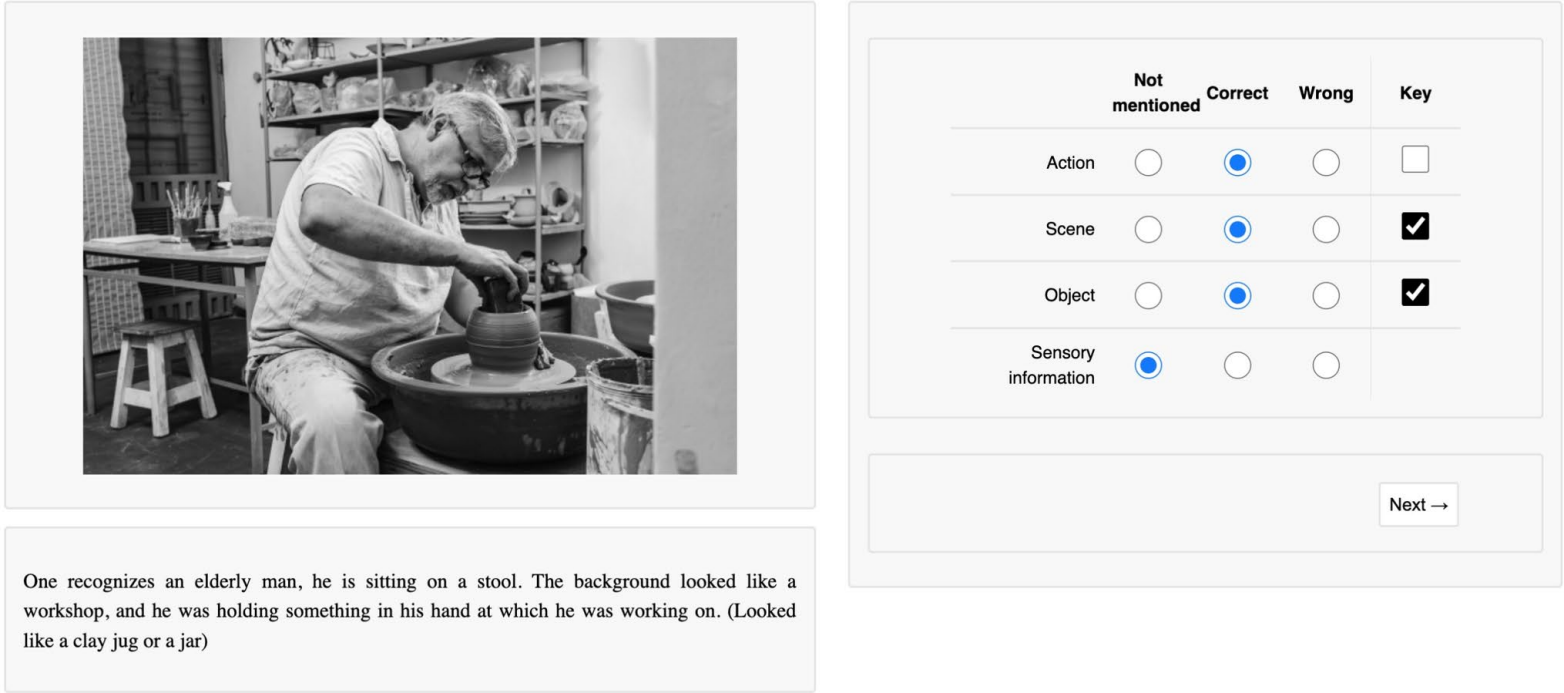
Example trial used in stage two (unlimited presentation time). Each trial consisted of an image and the corresponding description obtained from stage one (left side), and a checklist asking whether action, scene, object, and sensory information was mentioned in the description (right side). If a feature was not mentioned, raters were asked to tick “Not mentioned.” If a feature was mentioned, they should decide whether the described feature matched the image (“Correct”) or not (“Wrong”). The “Key”-box should be ticked for each feature separately if the key action, key scene, or the key object involved in the action was described correctly. Translated from German

During the first session, participants received written instructions regarding the experiment and how to run it at home using lab.js. (Henninger et al., 2022). To achieve high interrater reliability, participants performed nine practice ratings (not used for data analysis) under the guidance of an instructor (one of the authors, MR). Moreover, they performed the first batch in the first session and were encouraged to ask questions. Finishing one batch took approximately 1.5 to 2 h, and the whole rating lasted approximately 20 h per participant. In each trial, one of the descriptions obtained in stage one was presented together with the corresponding image and a checklist (see Fig. 2 for an example trial). In the checklist, participants were asked to rate if different features including the action, the scene, the object, and sensory information were present in the description, and if the description matched the image.

Each stimulus contained a very specific, goal-directed action and a very specific scene. For example, the image shown in Fig. 2 depicts a man “doing pottery” in a “workshop.” We referred to these specific actions and scenes as *key actions* and *key scenes*. Additionally, most images contained other actions that were less specific. For example, the man in Fig. 2 also “sits” on a chair, “holds” a jar, or “bends” his arm. In case descriptions contained several actions, we instructed our raters to evaluate whether most actions (irrespective of the level of specificity) were described correctly. The same was true for objects and scenes. As an example, for scenes, also unspecific answers, such as “inside,” should be labeled as correct descriptions. The exception to this rule was the object category “human body.” As a human was depicted at the center of each image, the ”Object” question was restricted to nonhuman objects. However, clothing items (e.g., trousers) were still considered objects.

Presumably, information about several unspecific actions needs to be integrated to recognize key actions. For example, recognizing someone “sitting” and “holding something” might facilitate identifying actions, such as “writing” or “doing pottery,” whereas it impedes recognizing actions, such as “running” or “climbing” (Lingnau & Downing, 2024). To distinguish between the stimulus presentation times that are required to recognize specific key actions and unspecific actions, we additionally asked the raters to indicate if the key action, the key scene, and the key object were described correctly. In the example shown in Fig. 2, the description mentioned that someone is “working at something.” The action “working” should be rated as correct, but the “Key” box should not be ticked, because the key action in this particular image is “doing pottery.” Key objects were defined as “objects involved in the action.” Participants were instructed to tick a separate box at each feature (“Key”) when they thought that the key feature was described correctly. Note that all raters were instructed to identify the key elements in every image by themselves, because we did not want to bias raters with specific labels. The instructions provided to the raters can be seen in Supplementary Material 4.

#### Comparison of the perceptual thresholds of action, object, and scene recognition

##### Fitting psychometric functions

Stage 2 resulted in five ratings for each feature per presentation time, image, and rater. Features included “Action”; “Scene”; “Object”; “Sensory”; “KeyAction”; “KeyScene”; and “KeyObject.” For each presentation time and rater, we counted how often a feature was rated as correctly described. For example, if the action “dancing” was correctly described (and thus, recognized) three times at PT = 50 ms, the count for the feature “action” at PT = 50 ms would be three. These counts were used to fit psychometric functions showing the probability of a correct feature description at each PT using the quickpsy package (Linares & López-Moliner, 2016) in R. For fitting the functions, the counts were collapsed across images and raters, leading to one count for each feature at each PT. Note that we did not fit a psychometric function for the feature “Sensory information,” because accuracy scores did not increase substantially with longer exposure durations. This is consistent with the results reported by Fei-Fei et al. (2007) and might have occurred, because participants did not describe vague shapes anymore once specific contents were recognized. For completion, we provide normalized accuracy scores for sensory information in Supplementary Material 2.

To account for differences between images with respect to complexity, we computed a normalization factor, following Fei Fei et al. (2007). To this aim, separately for each image and feature, we calculated the maximum score across presentation times. We later divided the collapsed counts by the sum of these maximum accuracy scores.

The lapse rate was calculated separately for each feature by using the proportion of correct responses in the 500-ms condition. Cumulative normal distribution functions were fitted to the data by using nonparametric bootstrapping (B = 1000) and maximum likelihood approximation. To evaluate the fit, the deviance and the Akaike Information Criterion (AIC) were calculated. The estimated parameters included the mean and the standard deviation of the psychometric function, as well as the guess rate defined as the probability of a correct response at zero stimulus intensity (Linares & López-Moliner, 2016).

##### Permutation testing

The resulting 50% thresholds were compared between features by using permutation tests. In each permutation (n = 1000), feature labels were randomly assigned to the data within presentation times. Next, curves were fit, and threshold differences between the permuted conditions were calculated. These randomly generated differences were then compared with the actual threshold differences obtained from the observed data. The resulting *p*-values represented the proportion of permutations in which the distance of the actual threshold pairs was smaller than the distance between the randomly generated threshold pairs (*α* = .05). Bonferroni-correction was applied to correct for multiple testing.

For the comparison between the 50% thresholds of scenes, objects, and actions, psychometric functions obtained from the features “Scene,” “Object,” and “Action” were compared. For the comparison of the 50% thresholds for the recognition of different action categories, we assumed that these categories represent specific key actions rather than unspecific actions (see also Supplementary Material 3). Therefore, data from the “KeyAction” feature was split by the five action categories, normalized as described above, and then used as input for the permutation tests.

#### Correlation of action, object, and scene recognition

##### Calculating accuracy scores

Following Fei-Fei et al. (2007), we calculated accuracy scores for each feature at each PT. To this aim, we divided the feature counts described above by the number of times each image was presented across participants in stage one, separately for each PT. The resulting ratio thus described how accurately a feature was described in each picture at a given PT, rated by an individual rater. For example, if the action “swimming” was correctly described three out of five times at PT = 66.6 ms, the resulting accuracy score would be 3/5 = 60%. Next, to account for different image complexities, we normalized accuracy scores within images by dividing them by the highest score achieved for the image across PTs (Fei-Fei et al., 2007). Note that this approach is similar to the normalization described in the section *Fitting Psychometric Functions*, with the difference that we did not sum up accuracy scores and maximum accuracies across images before normalizing. Finally, we averaged the normalized accuracy scores across raters, leaving one accuracy score for each feature at each PT for each image.

##### Correlation analysis

For each PT, we computed pairwise Pearson correlations between the normalized accuracy scores of the features “Scene,” “Object,” and “Action” across images. Pearson correlations were calculated using the SciPy library (Virtanen et al., 2020). Scatter plots were generated using the Python data visualization library Seaborn (Waskom, 2021) and Matplotlib (Hunter, 2007).

#### Exploratory analyses

We conducted four non-preregistered exploratory analyses. First, we investigated whether action specificity contributed to the speed of action recognition. To address this question, we compared 50% thresholds of the features “Action” and “KeyAction” using the technique described above.

Second, to determine whether the speed of recognition differs between more specific actions and scenes, we compared the 50% thresholds of the features “KeyAction” and “KeyScene.”

Third, to determine whether pairwise correlations between normalized accuracy scores for “Scene,” “Object,” and “Action” were statistically significant we employed a modified Z-test (α = 0.05), which accounts for dependent correlations and overlapping variables (Meng et al., 1992). This analysis was performed using “cocor.dep.groups.overlap” within the *cocor* R package (Diedenhofen & Musch, 2015).

Fourth, to determine whether stimulus complexity may have contributed to the differences in the presentation time that is required for the recognition of the five different action categories, we compared the stimulus complexity ratings obtained in the pilot study (Supplementary Material 1) between action categories. Twenty participants were asked to rate image complexity on a continuous scale ranging from low (0) to high (1). They were instructed to base their judgement on different image components, such as the background or the number of people depicted, and not to focus only on the depicted actions. A Friedman rank-sum test was calculated by using complexity ratings as dependent variable and action category as independent variable, followed by pairwise Wilcoxon signed-rank tests.

## Results

### Time course of action, object, and scene perception

#### Comparison of the Perceptual Thresholds of Action, Object, and Scene Recognition

**Fig. 3:**
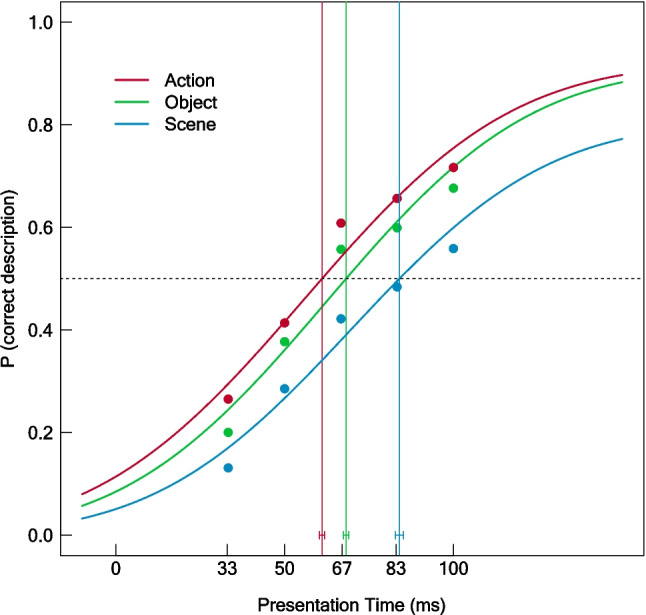
Psychometric functions for actions, objects, and scenes. Probability of a correct description of the action (red), object (green), and scene (blue) at each presentation time. Points were generated by averaging the proportion of correct feature descriptions across images and raters within presentation times. Vertical lines show the 50% thresholds. Error bars indicate the 95% confidence intervals for the 50% thresholds

To reveal the stimulus presentation times that are required for the recognition of actions, objects, and scenes, we fitted separate psychometric functions on the counts for the correct descriptions of these three features. The obtained psychometric functions and the corresponding 50% thresholds are depicted in Fig. 3 (see Table 1 for the 50% thresholds, confidence intervals, and fit indices). As shown, 50% thresholds for the recognition of actions were reached at the shortest presentation times (61.08 ms), followed by objects (68.22 ms) and scenes (84.01 ms). These observations were supported by the corresponding statistics. Two-sided permutation tests revealed a statistically significant lower threshold for action recognition than for object (*p* < .001) and scene (*p* < .001) recognition. Additionally, the recognition threshold for objects was significantly lower than the threshold for scenes (*p* < .001).

**Table 1:** Psychometric Thresholds for Actions, Objects, and Scenes

Feature	50% Threshold [ms]	95% CI	Deviance	*p*	AIC
Action	61.08	[60.30, 61.80]	9510.52	1	14407.69
Object	68.22	[67.45, 69.03]	10491.31	1	15065.55
Scene	84.01	[82.85, 85.19]	9913.52	1	14738.62

*Note*. Perceptual thresholds (50%) and 95% confidence intervals (CI) for actions, objects, and scenes. *P*-values represent the proportion of bootstrapping deviances higher than the empirical deviance. *P*-values > .99 indicate a good fit

### Exploratory Comparison of the 50% Thresholds for Actions and Key Actions

**Fig. 4:**
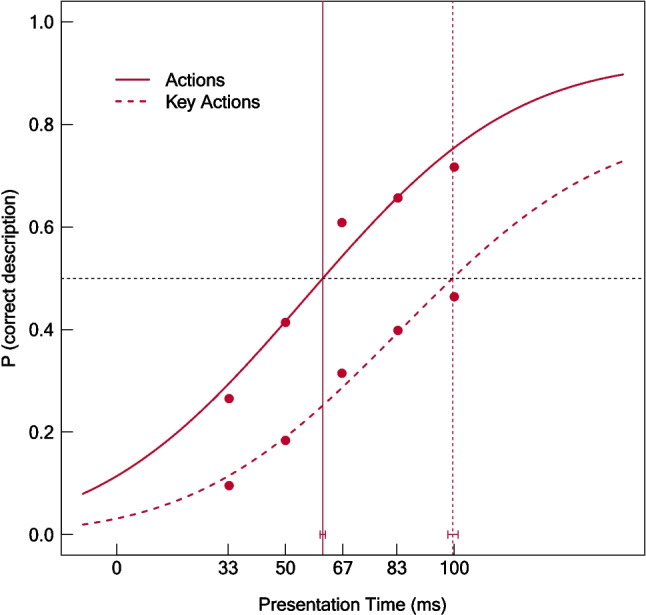
Psychometric functions for (general) actions (straight line) and (specific) key actions (dashed line). Points were generated by averaging the proportion of correct feature descriptions across images and raters within presentation times. Vertical lines show the 50% thresholds. Error bars indicate the 95% confidence intervals for the 50% thresholds

As stated, we assume that the recognition of specific key actions (such as “doing pottery”) relies on information regarding less specific actions, partially related to body postures (e.g., sitting, standing, holding). This implies that unspecific actions (which were included in the action feature) could be recognized at shorter presentation times than specific key actions. To test this, we compared the 50% threshold for the recognition of key actions with the threshold for the recognition of general actions. Note that we did not preregister this hypothesis, because we did not plan to distinguish between actions and key actions in our original analysis plan. The corresponding results are shown in Fig. 4. As evident, longer presentation times were required to recognize (specific) key actions (50% thresholds: 99.53 ms, 95% confidence interval [CI] [98.05, 101.08]) compared with (general) actions (50% threshold: 61.08 ms, 95% CI [60.30, 61.80]). Fit indices indicated a good fit for both curves (Action: Deviance = 9510.52, *p* = 1, AIC = 14407.69; Key Action: Deviance = 10203.62, *p* = 1, AIC = 14151.76). Two-tailed permutation tests showed that the difference between the two thresholds was statistically significant (*p* < .001).

### Exploratory comparison of the 50% thresholds between key actions and key scenes

**Fig. 5:**
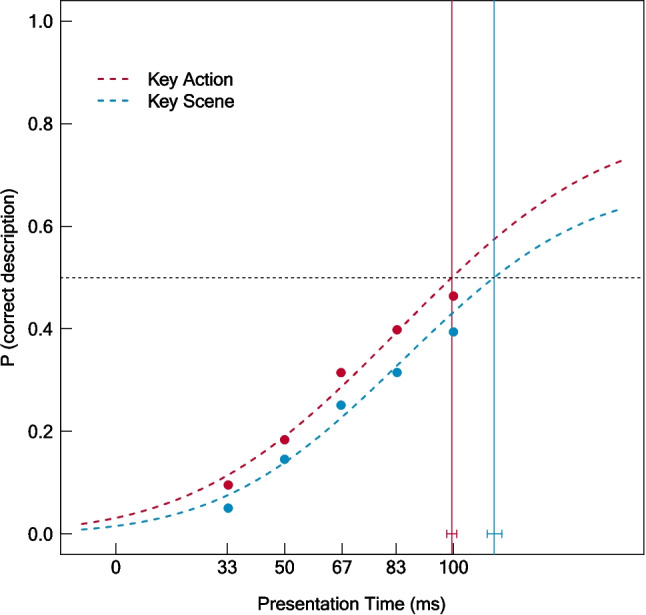
Psychometric functions for key actions (red) and key scenes (blue). Points were generated by averaging the proportion of correct feature descriptions across images and raters within presentation times. Vertical lines show the 50% thresholds. Error bars indicate the 95% confidence intervals for the 50% thresholds

In our first exploratory analysis, we found that action specificity affected the stimulus presentation time that is required for action recognition. To determine whether the differences obtained between stimulus presentation times required to recognize actions and scenes also hold if we focus on specific actions and scenes, we exploratively determined 50% thresholds for the features “KeyAction” and “KeyScene.” The resulting psychometric functions are depicted in Fig. 5. As shown, shorter presentation times were required to recognize key actions (50% threshold: 99.53 ms, 95% CI [98.01, 101.01]) compared with key scenes (50% threshold: 112.06 ms, 95% CI [109.97, 114.31]), in line with the results obtained for general actions and scenes shown in Fig. 3. Permutation testing revealed a significant difference between the 50% thresholds of key actions and key scenes (*p* < .001). The fit for both, key actions (Deviance = 10203.62, *p* = 1; AIC = 14151.76) and key scenes (Deviance = 8269.83, *p* = 1; AIC = 12144.69) was good.

### Correlation of action, object, and scene recognition

**Fig. 6:**
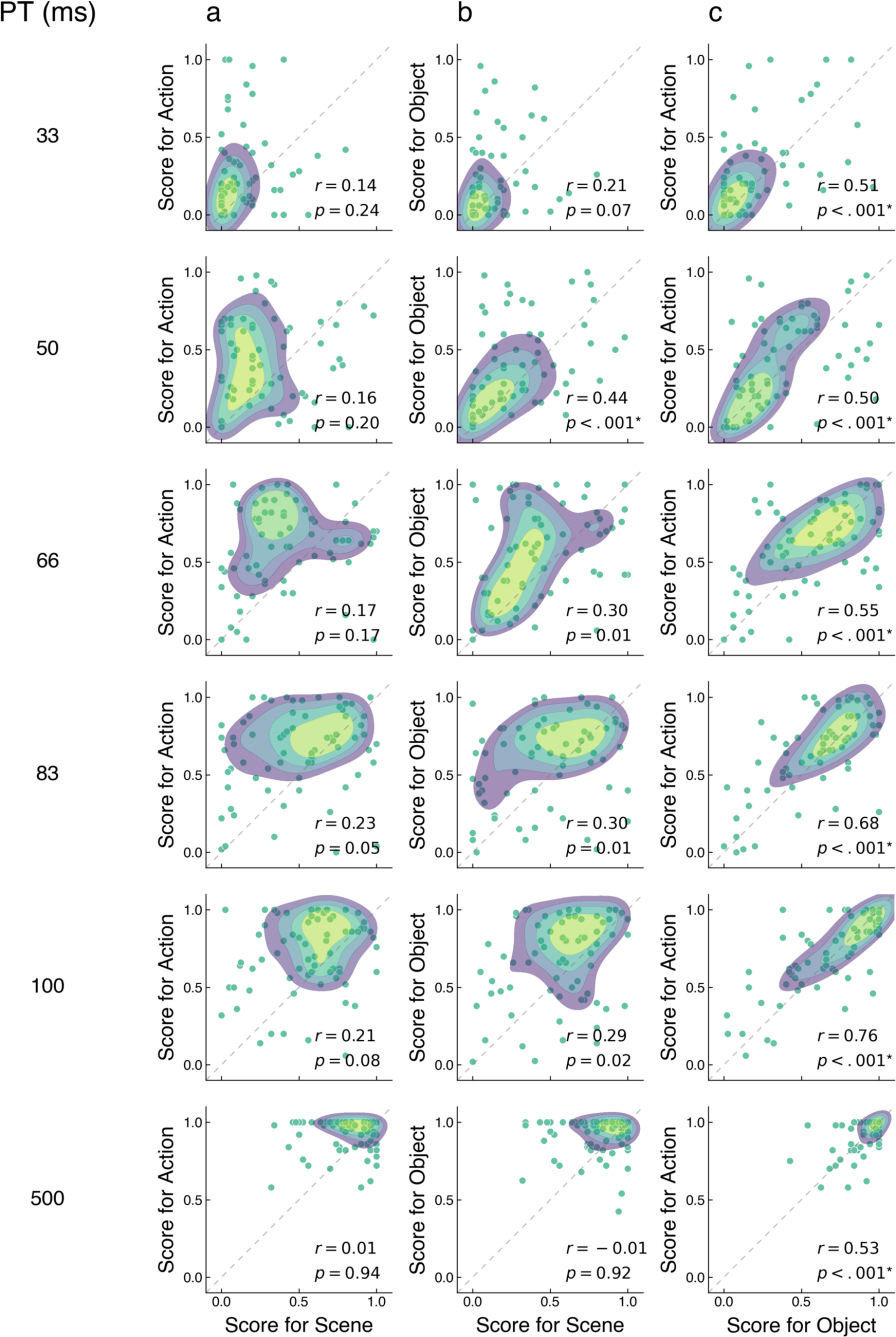
Pearson correlation between normalized accuracy scores for actions and scenes (**a**), objects and scenes (**b**), and actions and objects (**c**) across images, separately for each presentation time. Each dot represents the normalized accuracy score of one image along the two features. The color patches represent the density of accuracy scores within each scatter plot with more dense locations highlighted in brighter green patches. Overlaid are estimated correlation coefficients and *p*-values. *P*-values that survived Bonferroni correction (adjusted α = 0.003) are highlighted by an asterisk

To better understand the relationship between the recognition of actions, objects, and scenes, we computed pairwise correlations between the normalized accuracies for all three possible pairings of these three features for each PT across images. Figure 6 shows the resulting correlations. As shown, the accuracy for action recognition was significantly correlated with the accuracy for object recognition at all presentation times. Additionally, we found a significant correlation between the accuracies for scene and object recognition at PT = 50 ms. By contrast, across all presentation times, the recognition accuracies of actions and scenes were not significantly correlated. For completion, we repeated the correlation analysis between key features, and between key features and unspecific features, and obtained similar results (see Supplementary Material 5).

**Fig. 7:**
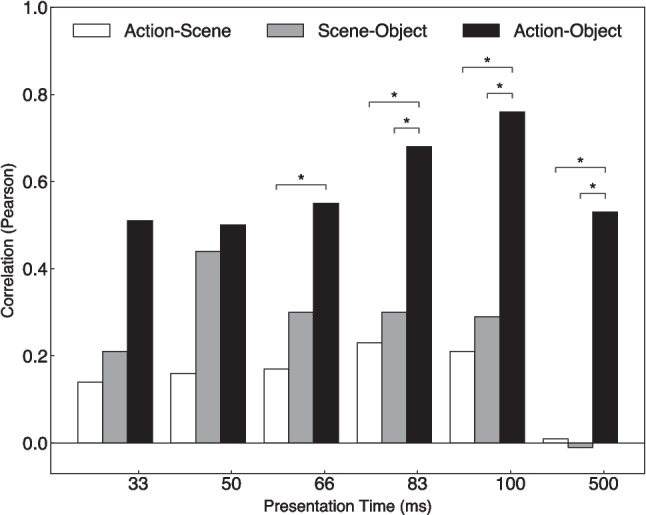
Pearson correlation of normalized accuracy scores between feature pairs across images, separately for each presentation time. Asterisks indicate significant differences between correlations of feature pairs after Bonferroni correction for multiple comparisons. Statistical comparison between dependent feature pairs correlations were obtained via the cocor package (Diedenhofen & Musch, 2015) using a modified Z-test (Meng et al., 1992)

Figure 7 shows direct comparisons of correlations between feature pairs. Exploratory Z-tests revealed that the pairwise correlations between actions and objects (black bars in Fig. 7) were significantly higher than the pairwise correlations between actions and scenes (white bars) from presentation times of at least 66 ms onwards, and higher than the pairwise correlations between scenes and objects (gray bars) at presentation times from 83 ms onwards. By contrast, pairwise correlations did not significantly differ between actions and scenes, and object and scenes, at any presentation time.

### Time course of recognizing different action categories

#### Perceptual thresholds of different action categories

**Fig. 8:**
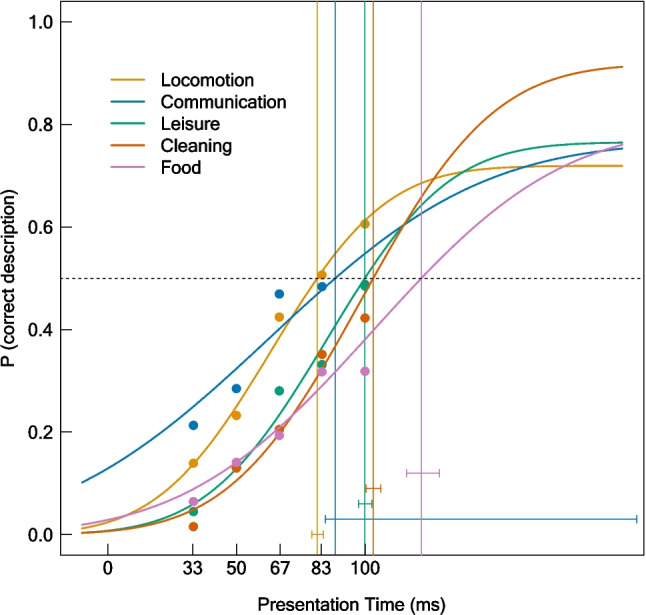
Psychometric functions for different action categories (Key Actions). Probability of a correct action description for the feature “KeyAction" at each presentation time, separately for each action category (“Locomotion,” “Communication,” “Leisure,” “Cleaning,” and “Food”). Points were generated by averaging the proportion of correct feature descriptions across images and raters within presentation times and action categories. Vertical lines show the 50% thresholds. Error bars indicate the 95% confidence intervals for the 50% thresholds

We additionally compared the 50% thresholds between different action categories. The psychometric functions for the different action categories are depicted in Fig. 8. Actions belonging to the superordinate category locomotion (50% threshold: 81.42 ms) required the shortest presentation times to be recognized, followed by actions related to communication (88.43 ms), leisure (99.93 ms), and cleaning (103.22 ms). Food-related actions (121.92 ms) required the longest presentation times to be recognized. The estimated thresholds and goodness of fit indices are reported in Table 2. Table 3 shows the results of the pairwise comparisons between the 50% thresholds of the different action categories. Similar results were found within an ANOVA reported in Supplementary Material 2.

**Table 2:** Psychometric Thresholds for Different Action Categories (Key Actions)

Category	50% Threshold [ms]	95% CI	Deviance	*p*	AIC
Locomotion	81.42	[79.27, 83.90]	2062.92	1	3022.77
Communication	88.43	[84.61, 105.68]	2356.93	1	3212.20
Leisure	99.93	[97.40, 102.68]	1791.56	1	2559.71
Cleaning	103.22	[100.44, 106.19]	1434.72	1	2149.10
Food	121.92	[116.14, 128.93]	1851.38	1	2525.87

*Note*. Perceptual thresholds for the five different action categories (key actions). 95% confidence intervals refer to the 50% thresholds. *P*-values represent the proportion of bootstrapping deviances higher than the empirical deviance. *P*-values > .99 indicate a good fit

**Table 3:** P-values of Pairwise 50% Threshold-Comparisons Between Action Categories (Key Actions)

	Locomotion	Communication	Leisure	Cleaning	Food
Locomotion		.111	<.001*	<.001*	<.001*
Communication			.037	.013	<.001*
Leisure				.392	<.001*
Cleaning					<.001*
Food					

*Note*. Two-sided *p*-values of the pairwise comparisons of 50% thresholds between basic level actions belonging to different superordinate action categories (see also Zhuang & Lingnau, 2022). *Statistically significant differences after Bonferroni correction (Bonferroni-corrected α = .005)

### Comparison of stimulus complexity between action categories

**Fig. 9:**
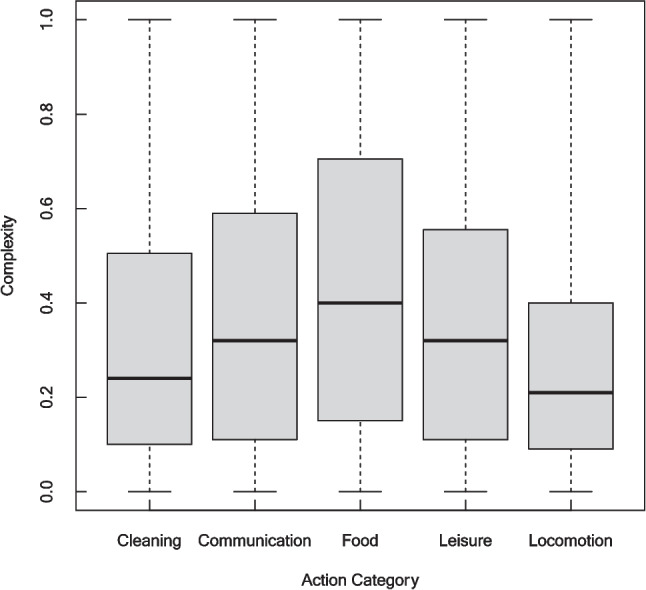
Complexity ratings for each superordinate action category. Boxes indicate the upper and lower quartile. Medians are shown by horizontal lines within the boxes. Whiskers depict the minimum and maximum complexity rating within each action category

The boxplots in Fig. 9 show the medians, upper quartiles, and lower quartiles of the complexity ratings, divided by action category. As shown, complexity ratings differed between action categories; the highest complexity ratings for actions belong to the superordinate category, food-related actions, and the lowest complexity ratings for actions belong to the category locomotion. These observations are supported by the corresponding statistics: the Friedman rank-sum test revealed significant complexity differences between the five action categories (χ^2^(4) = 28.50, *p *< .001). Post-hoc Wilcoxon signed-rank tests revealed significant differences between “Food” and “Cleaning” (*p* < .001), “Locomotion” and “Communication” (*p* = .016), “Locomotion” and “Food” (*p* < .001), and “Locomotion” and “Leisure” (*p* = .002). All *p*-values were Bonferroni-corrected for multiple comparisons.

## Discussion

We aimed to determine (1) the presentation time that is required to recognize actions, (2) objects and scenes, and (3) the degree to which the required presentation times differ between categories.

### Time course of action recognition

We found that action-related information could be accumulated rapidly, with stimulus presentation times as short as 60 ms. Recognizing key actions (e.g., doing pottery) required significantly longer presentation times (approximately 100 ms). These results generalize previous behavioral findings about rapid social interaction perception (Hafri et al., 2018) to other action categories.

Our results raise the question of how our brain extracts action-relevant information with such short presentation times. First, it is possible that participants’ attention is drawn toward the actor in a scene, which might explain the very fast processing of unspecific actions. This, in turn, may facilitate or impede the recognition of non-human objects and more specific actions, depending on the compatibility between these different sources of information. In fact, animacy is assumed to be processed in a privileged way (Kirchner & Thorpe, 2006; Thorpe et al., 1996), which is particularly strong for faces (Bindemann et al., 2005) and bodies (Downing et al., 2004). Future studies are required to test these predictions more systematically.

Another possible explanation for the short stimulus presentation times that are required for action recognition is that rapidly extracted low-spatial frequency information might be sufficient to identify body postures. In fact, the orientation of bodies has been shown to be extracted within the first 30 ms of stimulus presentation (Glanemann et al., 2016). It is possible that such information is sufficient to activate representations of actions, which are strongly associated with certain body postures (see also Papeo & Abassi, 2019). This might be particularly true for unspecific actions, such as “sitting,” which is not associated with specific objects or scenes. Still, scene and object information might be important to distinguish between more specific actions, e.g., “writing” (in an office). This is supported by our exploratory results, showing that participants required longer presentation times to recognize specific key actions compared with unspecific actions.

### Time course of action, object, and scene recognition

Bar (2004) suggested that rapidly extracted low-spatial frequency information conveys vague shape information that activates probable context frames (even when the scene is not yet fully analyzed). Both then activate representations of probable objects, and correctly perceived objects can in return facilitate scene recognition (see also Davenport & Potter, 2004). Does a similar bidirectional relationship also hold for scenes and actions, and for objects and actions? In other words, does scene and/or object information contribute to action recognition even if the scene and/or action is not yet fully analyzed (see also Lingnau & Downing, 2024)? In fact, scene and object information have been suggested to contribute to action recognition (El-Sourani et al., 2018; Wurm et al., 2012; Wurm & Schubotz, 2017). The fact that the recognition of specific key actions in the current study required presentation times of approximately 100 ms, whereas thresholds for the recognition of objects (68 ms) and scenes (84 ms) were reached earlier, with even lower thresholds for unspecific actions (<60 ms) compatible with this view. Our correlation analysis further suggested that object and action information either facilitate each other or share computational resources, in line with the proposal of a strong connection between object and action recognition (Bach et al., 2014; El-Sourani et al., 2018; Kalénine et al., 2016; Mounoud et al., 2007). By contrast, we found no such relationship between actions and scenes irrespective of the level of specificity (see Supplementary Material 5 for correlations between key actions and key scenes). In fact, additional Z-tests revealed stronger correlations between accuracy scores for object and action information than between object and scene information at all presentation times longer than 66 ms, i.e., those presentation times that are sufficient for the recognition of action information.

It should be noted that our paradigm is not suitable to determine how long participants needed to process the information related to actions, objects, and scenes after stimulus presentation (for a discussion of the differences between presentation time and processing time, see VanRullen, 2011). Likewise, we did not investigate the order in which the brain processes action, object, and scene information. Instead, the focus of the current study was to determine how long a stimulus needs to be presented to enable successful processing of actions, objects, and scenes. We found that presentation times of approximately 60 ms are sufficient for action recognition and that shorter presentation times are needed for recognizing unspecific actions reflecting static body posture than objects when both elements are present in a scene. This finding may seem at odds with models suggesting that object information plays a key role in recognizing other people’s actions (Bach et al., 2014; Lingnau & Downing, 2024; Wurm et al., 2012). That said, given that bodies/faces are known to attract attention and thus are likely to draw attention away from objects, it is possible that presentation times do not differ for recognizing unspecific actions and objects when these are presented separately. Moreover, because our data do not speak to the question in which order the brain processes action, object, and scene information, it is still possible that object-relevant information gathered in that time window contributes to recognizing the action, even if the object has not been fully recognized yet. Moreover, we would like to point out that we compared the perceptual thresholds for recognizing actions and objects at a general level, regardless of whether the objects were part of the action. Comparing recognition thresholds for actions with and without objects would be interesting, but our study was not designed to address this question as most stimuli depicted actions involving objects.

Different paradigms are required to address how much time is required to process information related to actions, objects, and scenes. For example, combining EEG data with model-based representational similarity analysis (RSA), it has been shown that patterns of EEG activity show the highest similarity with models capturing action-related features (e.g., transitivity) around 170 to 345 ms after stimulus onset, indicating action-related processing within this time window (Dima et al., 2022).

Our results are in line with the view that perceptual evidence for actions, objects, and scenes is accumulated in parallel and that object and action recognition can facilitate each other (see also Kalénine et al., 2016; Mounoud et al., 2007). However, scene information might be less important for recognizing actions than object information. Wurm & Schubotz (2017) suggested that the recognition of actions profits from scene information when information about the action is sparse, which might not have been the case in the current study. Further experiments are required to determine the circumstances under which scene and action information impact each other.

### Time course of action recognition for different categories

We obtained that the 50% thresholds for specific actions belonging to the categories “Locomotion” and “Communication” required the shortest exposure durations (81 and 88 ms, respectively), whereas food-related actions required the longest exposure durations (approximately 120 ms). The low perceptual thresholds of communication-related actions (e.g., “talking,” “arguing,” and “punching”) are in line with previous studies suggesting that social interactions can be recognized rapidly (Hafri et al., 2018; McMahon & Isik, 2023). By contrast, we did not expect particularly fast accumulation of information for actions related to “Locomotion.” One might argue that some actions belonging to this category are particularly evolutionary relevant (e.g., “running” or “climbing”) and that it might be more urgent to determine that someone is running away from a threat than to figure out what another person is about to eat.

While most food-related actions were performed in kitchens or restaurants, many locomotion-related actions were depicted outside in front of rather plain backgrounds. Thus, one may wonder whether stimulus complexity might have contributed to the obtained differences. Our complexity ratings (see Supplementary Material 3) revealed that images belonging to the food category were indeed rated to be more complex than images belonging to other categories. We took these differences into account by normalizing the accuracy scores by the maximum accuracy score obtained for a given image and feature, which should have diminished possible effects of image complexity on our data. Further experiments are required to tease apart the effects of action category and stimulus complexity more systematically.

## Limitations and conclusions

The experiments presented have several limitations. First, because we aimed to keep our stimuli as naturalistic as possible, we did not control for low level visual features, such as the size and eccentricity (position relative to image center) of the actors’ bodies, faces, and hands, or the size of the objects involved in the action during stimulus selection. Actors and objects might be depicted prominently in the image center, which might have selectively enhanced action and object recognition compared to scene recognition. To address this concern, we retroactively manually determined the size and eccentricity for the hands, bodies, and objects involved in the action for every image. Eccentricity estimates show that both objects involved in the action (“Key Object”) and bodies were well distributed across the images, tending to be depicted slightly off center (Fig. S8). Furthermore, bodies tended to vary in size, whereas both objects involved in the action and hands manipulating them were typically small (Fig. S9). To find out whether eccentricity and size of these items differentially affect accuracy scores for action, objects, and scenes, we ran a linear regression using eccentricities and relative sizes of bodies, hands and objects of the action to predict accuracy scores (see Supplementary Material 6). We found no significant differences in coefficients between actions, objects, and scenes. These results argue against a selective enhancement of action and object recognition because of their eccentricity and relative size in the images of this stimulus set. To enable a more direct comparison of presentation times required for the recognition of actions, objects, and scenes, future studies should use stimuli in which size and eccentricity of hands, bodies, and objects involved in the action and scene focus are matched and in which these elements are either shown in parallel or in isolation.

Second, one may wonder to which extent our results generalize to dynamic stimuli. Dynamic stimuli provide information, such as movement kinematics, that is important for action recognition under certain circumstances (Lingnau & Downing, 2024). Static images provide a rich source of information even with respect to dynamic information (Freyd & Finke, 1984), and brain regions known to preferentially respond to dynamic stimuli have been shown to respond to static images of human actors implying motion (Kourtzi & Kanwisher, 2000). A wide set of brain regions has even been shown to represent actions across static and dynamic stimuli (Hafri et al., 2018). Moreover, the use of dynamic stimuli, where the available information unfolds in time, can be problematic when the goal is to determine perceptual thresholds or the temporal evolution of the underlying brain signatures. Consequently, it is relatively common to use static images in behavioral (Fei-Fei et al., 2007; Zhuang & Lingnau, 2022) and neuroimaging studies (Kabulska et al., 2024; Tucciarelli et al., 2019; Zhuang et al., 2023) on action recognition. In sum, while it will be interesting for future studies to determine whether perceptual thresholds differ between static and dynamic stimuli, the use of static images in the current study provides a valuable approach for investigating these processes.

Third, to stay as closely as possible to the original study by Fei-Fei et al. (2007), we used images in grayscale. Color information has been suggested to contribute to the recognition of scenes (Castelhano & Henderson, 2008) and objects (Witzel & Gegenfurtner, 2018). We thus cannot exclude that we selectively impeded scene and object recognition by removing color information. Conversely, other authors argued that color information might not be important for rapid scene categorization (Delorme et al., 2000; Fei-Fei et al., 2005). It thus will be interesting for future studies to examine how well the results of the current study generalize to colored images.

In summary, we showed that in the presence of both actions and objects within a scene, information necessary to recognize unspecific actions such as sitting or standing can be extracted with exposure duration as short as 60 ms, while slightly longer exposure durations are required to extract object- and scene-related information, and even longer exposure durations for more specific actions. We additionally found that shorter exposure durations are required to recognize locomotion and communicative actions than food-related actions. Together, our results are in line with the view that information about objects, scenes, and unspecific actions is gathered rapidly and in parallel and that evidence from objects and actions is integrated until a certain evidence threshold in favor of a specific action is exceeded (Lingnau & Downing, 2024).

## Supplementary information

Below is the link to the electronic supplementary material.

Supplementary file1 (PDF 86 KB)

Supplementary file2 (PDF 214 KB)

Supplementary file3 (PDF 478 KB)

Supplementary file4 (PDF 775 KB)

Supplementary file5 (PDF 135 KB)

Supplementary file6 (PDF 749 KB)

## Data Availability

All primary data are publicly available via the following link: https://osf.io/u3p5t/?view_only=4aa7c80c734046c2b04f8c53c9a9d3d9 All study materials are publicly available via the following link: https://osf.io/u3p5t/?view_only=4aa7c80c734046c2b04f8c53c9a9d3d9

## Footnotes

1 Link to our OSF repository: https://osf.io/u3p5t/?view_only=4aa7c80c734046c2b04f8c53c9a9d3d9

## Code availability

All analysis scripts are publicly available via the following link: https://osf.io/u3p5t/?view_only=4aa7c80c734046c2b04f8c53c9a9d3d9
