# Supplementary material for: Actions at a glance: The time course of action, object, and scene recognition in a free recall paradigm
Source: Cogn Affect Behav Neurosci. 2025 Feb 26;25(3):693–707. doi: 10.3758/s13415-025-01272-6 (PMC12130074; doi:10.3758/s13415-025-01272-6)
Supplement: Supplementary file 1 — Supplementary file1 (PDF 86 KB) [file 13415_2025_1272_MOESM1_ESM.pdf]

Supplementary Material 1

Table S1

Preregistration deviations

#	Details		Original wording	Deviation description	To what extent is this a deviation from the preregistered plan?	Judgment of impact
1	Type	Methods	*Each stimulus will be presented once to each participant, and presentation times will be balanced across participants.*	*Each stimulus was presented once to each participant. However, similar to Fei-Fei et al. (2007), presentation times were assigned randomly to each participant, and balanced across images.*	Minor	*We do not expect this change to have a significant impact on the outcome of this study as we did not analyze data at an individual level. Instead, we fitted psychometric functions on all datapoints, accumulated across participants.*
	Reason	Miscommunication				
	Timing	Before data collection				
2	Type	Analyses	*Additionally, each attribute will be split into sub-attributes (e.g. scenes will be split in indoor vs. outdoor). The sub-attributes regarding sensory information, scene and objects are derived from Fei-Fei et al. (2007), and the action categories generated in stage 0 will build the sub-attributes of the action-attribute.*	*We noticed that the rating experiment already took approximately 15-20 hours per rater. Thus, we decided to not include these additional questions. Instead, we added questions referring to key actions, key scenes, and key objects.*	Minor	*We aimed to collect these additional features to address exploratory questions. They were not necessary to address any of our preregistered hypotheses.*
	Reason	Plan not possible				
	Timing	During data collection				
3	Type	Analyses	*The main dependent variable will be the description accuracies for a set of pre-defined attributes from free-recall descriptions of each stimulus at each*	*As we did not include the key action feature in our preregistration, we did not preregister that we would use the key action data for comparing the recognition*	Minor	*We argue that the feature 'key action' is better suited than the preregistered feature 'action') since the action categories (and the corresponding actions)*
	Reason	New knowledge				
	Timing	During data				

		collection	*presentation time. Those attributes include sensory information, scene, objects, and the type of action presented in each picture. Additionally, each attribute will be split into sub-attributes (e.g. scenes will be split in indoor vs. outdoor). The sub-attributes regarding sensory information, scene and objects are derived from Fei-Fei et al. (2007), and the action categories generated in stage 0 will build the sub-attributes of the action-attribute.*	*speed of actions belonging to different categories.*		*selected for this study were based on a multi-arrangement task in which participants were instructed to judge the similarity of actions at the level of key actions (e.g. 'pottering') rather than less specific actions (e.g. 'sitting').*
4	Type	Analyses	*H1 & H2: A within-subject ANOVA will be conducted using presentation time (levels: 33.3, 50.0, 66.6, 83.3, 100, 500ms) and attribute (levels: sensory vs. scene vs. object vs. action) as within-subject factors.*<br><br>*H3: A within-subject ANOVA will be conducted using presentation time (levels: 33.3, 50.0, 66.6, 83.3, 100, 500ms) and action category (levels obtained from Stage 0) as within-subject factors.*	*After careful consideration, we decided that fitting psychometric functions is better suited for our research questions since this approach enables a direct comparison of perceptual thresholds between actions, objects, and scenes. This decision was made before any data from the main experiment were analyzed.*	Major	*To test whether this major deviation affected our results, we conducted the preregistered ANOVAs as well and report the results in Supplementary Material 4. The results were qualitatively similar to the results obtained by analysis based on psychometric functions.*
	Reason	New knowledge				
	Timing	After data access				

Note. Deviations from the preregistration.
