# Supplementary material for: Actions at a glance: The time course of action, object, and scene recognition in a free recall paradigm
Source: Cogn Affect Behav Neurosci. 2025 Feb 26;25(3):693–707. doi: 10.3758/s13415-025-01272-6 (PMC12130074; doi:10.3758/s13415-025-01272-6)
Supplement: Supplementary file 2 — Supplementary file2 (PDF 214 KB) [file 13415_2025_1272_MOESM2_ESM.pdf]

Supplementary Material 2

Preregistered Analyses that were not reported in the main manuscript

Within-Subject-ANOVA: Comparison Between Action-, Object-, and Scene Recognition

To reveal the stimulus presentation times required for the recognition of actions, objects and scenes, we pre-registered an ANOVA with Presentation Time (levels: 33, 50, 67, 83, 100, 500 ms) and Feature (levels: action, object, scene, and sensory information) as within-subject factors. The inputs to the analysis were normalized accuracy scores which account for image complexity, as described in the Methods section, Stage 2, Secondary Analyses.

As can be seen in **Figure S1**, all features but the sensory information were described more accurately with longer exposure durations (main effect of presentation time: $F(5, 345) = 195.61, p < 0.001$, partial $\eta^2 = 0.74$; interaction Feature * Presentation Time: $F(15, 1035) = 35.36, p < 0.001$, partial $\eta^2 = 0.34$). Furthermore, the accuracy of the descriptions differed between the features (main effect of feature: $F(3, 207) = 64.92$, $p < 0.001$, partial $\eta^2 = 0.48$). Post hoc dependent t-tests indicated that the mean score for actions ($M = 0.59$, $SD = 0.32$) and for objects ($M = 0.55$, $SD = 0.34$) was significantly higher than the score for scenes [($M = 0.44$, $SD = 0.32$); action - scene $t(419) = 9.56$, $p < 0.001$; object – scene $t(419) = 7.23$, $p < 0.001$]. Additionally, the mean score for sensory information ($M = 0.26$, $SD = 0.15$) was significantly lower than for actions, objects and scenes combined [($M = 0.53$, $SD = 0.28$); $t(419) = -18.39$, $p < 0.001$].

From visually inspecting **Figure S1**, the Feature * Presentation Time interaction is driven by the accuracy scores for the feature ‘sensory information’, which shows no modulation by presentation time (in contrast to the accuracy scores for the remaining

features). To support this claim, and to draw a parallel to the main threshold analysis (**Figure 3**), we performed an additional ANOVA where we excluded scores for sensory information. As expected, the accuracy scores differed between features (main effect of Feature: $F(2, 138) = 16.43$, $p < 0.001$, partial $\eta^2 = 0.19$) and varied with presentation time (main effect of Presentation Time: $F(5, 345) = 206.54$, $p < 0.001$, partial $\eta^2 = 0.75$), but the difference between the actions, objects and scenes did not vary as a function of presentation time (interaction Feature * Presentation Time: $F(10, 690) = 0.96$, $p > 0.1$).

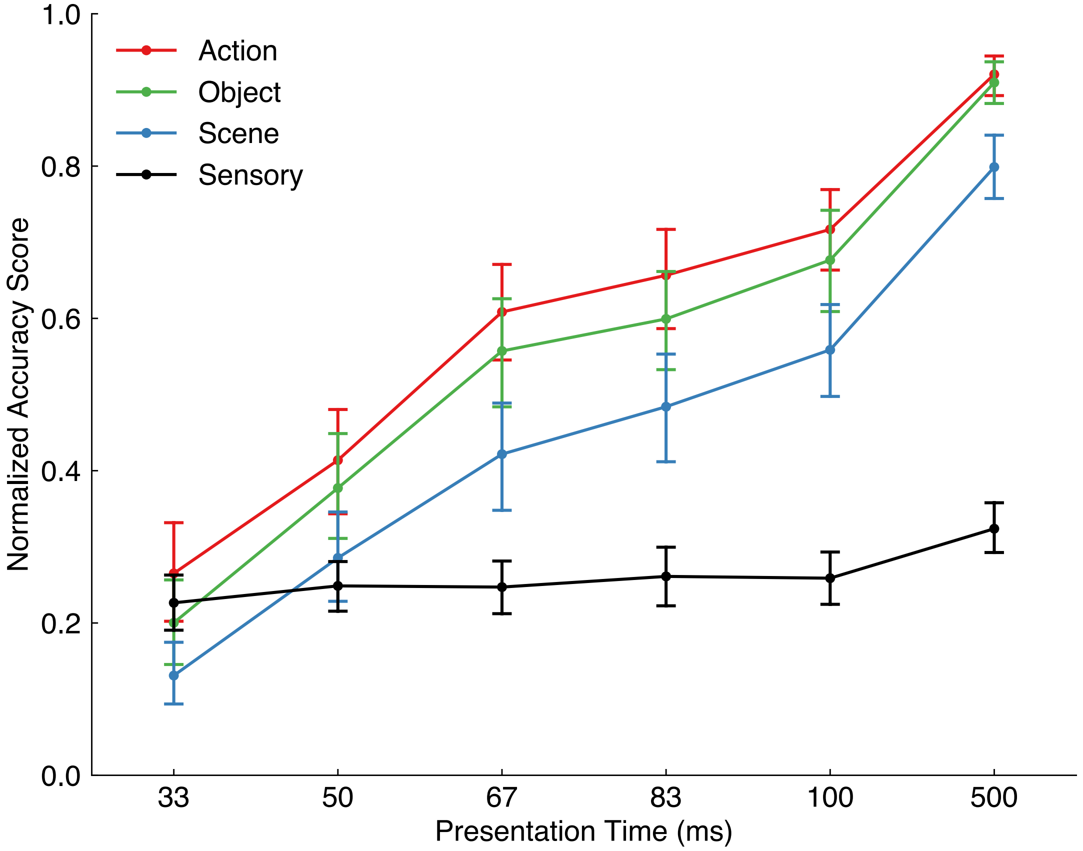

Figure S1. *Normalized description accuracy scores for each feature as a function of presentation time. Error bars indicate the 95% CIs of the means.*

Within-Subject-ANOVA: Comparison Between Different Action Categories

To reveal whether the speed of information accumulation for action recognition differs between action categories, we pre-registered a within-subject ANOVA using presentation time and action category as within-subject factors.

The inputs to the analysis were normalized accuracy scores of key actions (see **Figure 8**). As can be seen in **Figure S2**, the accuracy of image descriptions increased with longer exposures across action categories (main effect of presentation time: $F(5, 45) = 497.15$, $p < 0.001$, partial $\eta^2 = 0.98$). Accuracies differed between action categories, and this effect interacted with presentation time (main effect of action category: $F(4, 36) = 22.81$, $p < 0.001$, partial $\eta^2 = 0.71$; interaction Presentation Time * Category: $F(20, 180) = 35.70$, $p < 0.001$, partial $\eta^2 = 0.80$). Overall, these results are in line with our results from the threshold analysis reported in the main text.

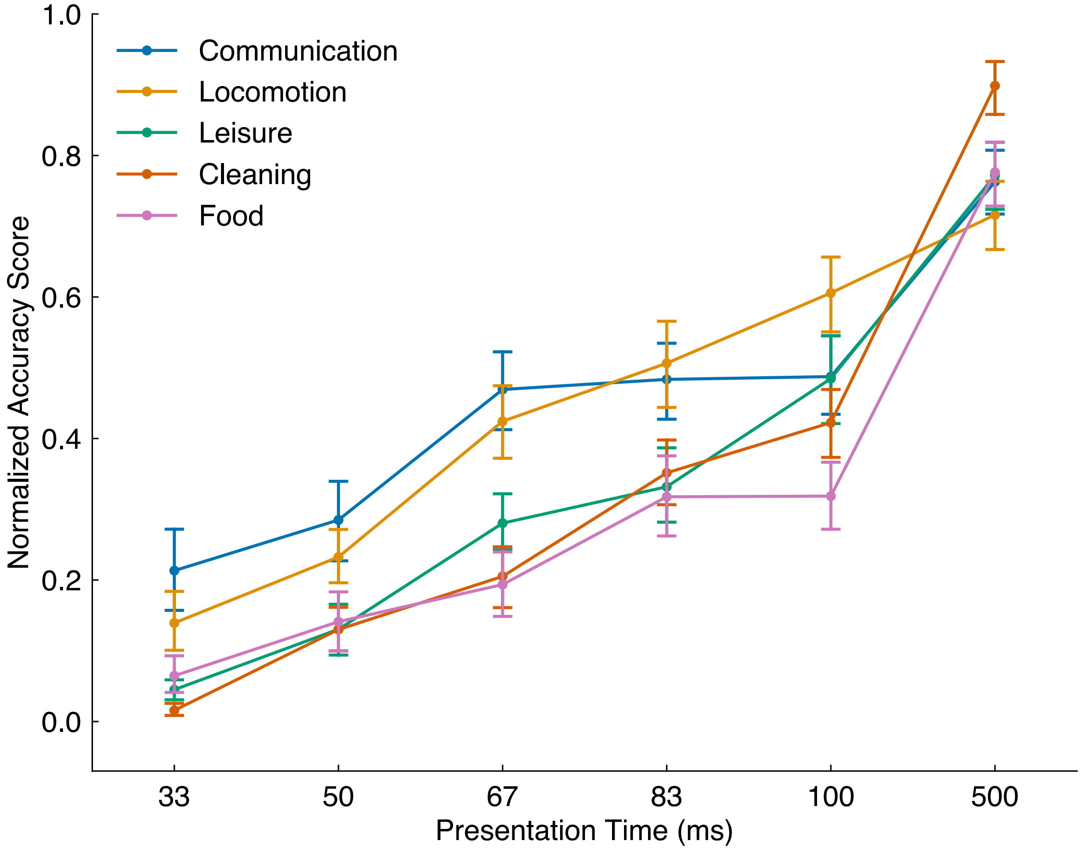

Figure S2. *Specific action description accuracy scores per action category as a function of presentation time. Error bars indicate the 95% CIs of the means.*

Multiple Linear Regression: Effects of Stimulus Complexity on Recognition Accuracy

To identify the impact of stimulus complexity (stimulus complexity ratings obtained in the pilot study, see **Supplementary Material 3** and **Figure 9**) on action recognition, we pre-registered and performed a linear regression analysis with presentation time and scene complexity ratings as independent variables, and description accuracy scores of actions (not normalized) as dependent variable. The regression was fit onto averaged accuracy scores across raters. The results of the regression analysis indicated that the two predictors and their interaction explained 43% of the variance (R^2_{adj} = 0.43, $F(3, 416)$ = 104.52,

$p < 0.001$). We found that presentation time and stimulus complexity significantly predicted the action description accuracy scores: $\beta_{PT} = 0.11$ ($t = 4.45$, $p < .001$), $\beta_{Complexity} = –0.63$ ($t = -2.47$, $p < .05$), $\beta_{Interaction} = 0.03$ ($t = 0.48$, $p = .63$), $\beta_{Intercept} = 0.40$ ($t = 4.18$, $p < .001$).

As expected, the complexity of presented scenes impeded action recognition, with actions being described less accurately when depicted in more complex scenes. Thus, even though this effect is quite small, these results reinforce our decision of normalizing accuracy scores.
