# Supplementary material for: Actions at a glance: The time course of action, object, and scene recognition in a free recall paradigm
Source: Cogn Affect Behav Neurosci. 2025 Feb 26;25(3):693–707. doi: 10.3758/s13415-025-01272-6 (PMC12130074; doi:10.3758/s13415-025-01272-6)
Supplement: Supplementary file 3 — Supplementary file3 (PDF 478 KB) [file 13415_2025_1272_MOESM3_ESM.pdf]

Supplementary Material 3

Pilot Experiment: Identifying Action Categories Within the Stimulus Set

For the main experiment, we aimed to present participants with an equal number of actions from several different action categories. We therefore decided to select no less than 10 actions from each action category. To be able to select action stimuli that are judged to belong to specific action categories by human observers, we conducted a multi-arrangement experiment (Kriegeskorte & Mur, 2012).

Methods

Participants

20 participants (2 male, 18 female) took part in this experiment, recruited via the online platform Sona Systems (https://www.sona-systems.com) and direct advertisement in WhatsApp group chats. To take part in the study, participants had to be German native speakers who were at least 18 years old. Additional prerequisites were normal or corrected to normal vision, and no diagnosed neurological diseases. Participants' age ranged from 18 to 34 years, with a mean age of $M = 21.68$ years ($SD = 3.79$ years). One participant did not report their age. All participants were enrolled as students at the time of the experiment, with 13 participants studying Psychology. All participants provided written informed consent prior to taking part in the study.

Stimulus Selection

The stimulus material consisted of 100 static images of human agents performing actions (e.g. brushing teeth, eating with chopsticks, or dancing) in a variety of natural contexts (e.g. bathroom, restaurant, or stage). We selected stimuli from the action categories revealed by Tucciarelli et al., 2019, namely “Cleaning”, “Communication”, “Food-related”,

“Leisure”, and “Locomotion”. Stimuli were acquired from the BU101 dataset (Ma et al., 2015), the HICO dataset (Chao et al., 2015), Shutterstock, and the Moments in Time Dataset (Monfort et al., 2020). We used the following criteria (see also Kabulska & Lingnau, 2022): (1) the performed action had to be the main aspect of the image; (2) the action had to be depicted in front of a naturalistic background; (3) the image needed to be taken in landscape orientation. Additionally, pictures were discarded if their background was blurred as this might have impeded scene recognition selectively. All stimuli were resized, cropped, and gray scaled using a custom python-based pipeline.

Materials

To ensure that individual stimuli in the multi-arrangement task were large enough to be clearly recognizable, we used a television screen (Samsung model UE49NU7179, 49 inches diagonal, UHD (3840 x 2160), screen refresh rate 60 Hz) in a laboratory at the University of Regensburg. All experiments belonging to the pilot experiment were conducted using Meadows, an online platform for behavioral experiments (http://meadows-research.com), running in a Microsoft Edge Browser (Version 108.0.1462.76) on an MS Windows 10 System. For the multi-arrangement task, the evidence utility exponent was kept at 10, and the evidence weight was set to 0.37 (following Kabulska & Lingnau, 2022). Additionally, we used a time limit of 90 minutes to avoid exhaustion of participants.

Procedure

First, participants gave informed consent to take part in the study and filled out a demographic questionnaire. Next, we carried out a multi-arrangement experiment (Kriegeskorte & Mur, 2012). In brief, in each trial, participants were presented with varying number of stimuli and were asked to arrange the actions on the screen via mouse drag and

drop inside a white circle according to the perceived similarity of the presented actions. As an example, an image depicting the actions eating and drinking would be expected to be positioned closer to one another than the actions eating and running. Other aspects of the image such as the background, the number of people performing the action, or the overall composition of the image should be ignored. In the first trial, all 100 stimuli had to be arranged within the circle, while in all subsequent trials, the subset was determined based on an adaptive algorithm (see Kriegeskorte & Mur, 2012, for details). The experiment finished once the desired evidence weight was reached, or if the time limit was exceeded. Before starting the task, a practice trial was performed in which pictures of five cats had to be arranged according to their similarity.

After completing the multi-arrangement experiment, participants were asked to rate all stimuli regarding their complexity. Here, each stimulus had to be rated separately on a continuous scale ranging from “low” (0) to “high” (1) complexity. At the end of the experiment, participants were compensated with course credits and sweets. The overall procedure took approximately two hours per participant.

Data Analysis

Data obtained from the multi-arrangement experiment were converted to individual representational dissimilarity matrices (RDMs), separately for each participant. These RDMs contained Euclidean distances measuring the pairwise dissimilarities between all depicted actions. RDMs were further analyzed using a custom python-based pipeline. First, RDMs were rescaled by the maximum value within participants and then collapsed across participants, resulting in one group RDM. Next, the group RDM was normalized to scale

the pairwise dissimilarities between zero and one. Following this, a metric multidimensional scaling (MDS) was performed.

To identify clusters according to which participants arranged the stimuli, we used hierarchical clustering, following Tucciarelli et al. (2019) and Kabulska & Lingnau (2022). To identify the best linkage method for the hierarchical clustering, the cophenetic correlation coefficients were calculated for different linkage methods. The highest scoring linkage method was "average" (cophenetic correlation = 0.769). Afterwards, to reveal how many clusters described the multi-arrangement data best, we computed the silhouette index (*si*) (Rousseeuw, 1987) for each cluster solution, ranging from 5 to 50 clusters. Finally, hierarchical clustering was performed by using the *AgglomerativeClustering* function from scikit-learn (Pedregosa et al., 2011).

Since the main goal of this pilot experiment was to select no less than 10 images per action category, after computing the silhouette indices we excluded all clusters that consisted of less than 10 images. Additionally, to gain equally sized action categories for the further procedure, all clusters were reduced to the size of the smallest remaining cluster. Cluster size was reduced by keeping the nearest stimuli to each cluster's centroid, based on shortest Euclidean distances.

Results

The analysis of the silhouette indices suggested an optimal cluster solution of five clusters ($si = 0.24$). However, one of these clusters would have been removed because it contained less than ten actions, leaving only four clusters. Therefore, other cluster solutions with high silhouette indices were also considered. **Figure S3** shows the silhouette indices for each cluster solution between five and 50 clusters. The main goal of the pilot experiment

was to select at least 10 actions from several different action categories within the stimulus set. This was achieved best by the ten-cluster solution which still had a comparably high silhouette index (*si* = 0.18).

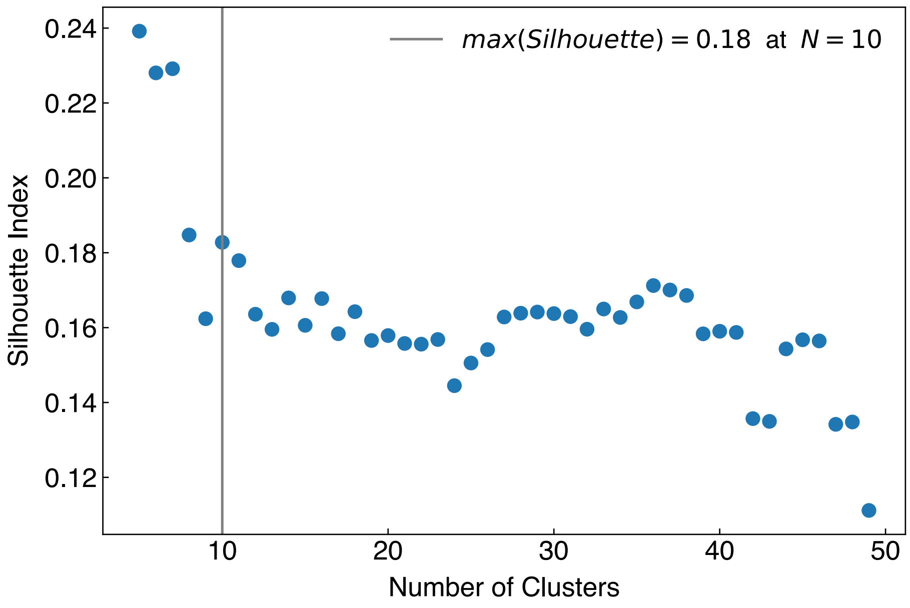

Figure S3. *Silhouette indices for cluster solutions between five and 50 Clusters. The grey line marks the silhouette index of the chosen ten-cluster solution.*

Figure S4 shows the names of all actions presented in the pilot experiment. After all clusters with less than ten actions were removed, five clusters remained. These five clusters corresponded to the action categories "Cleaning", "Communication", "Food-related", "Leisure", and "Locomotion", similar to the categories identified by Tucciarelli et al. (2019). The smallest of these clusters ("Leisure") contained 14 actions. Therefore, all action

categories were pruned down to 14 actions. At the end of the pilot experiment, 70 out of 100 images remained for the main experiment.

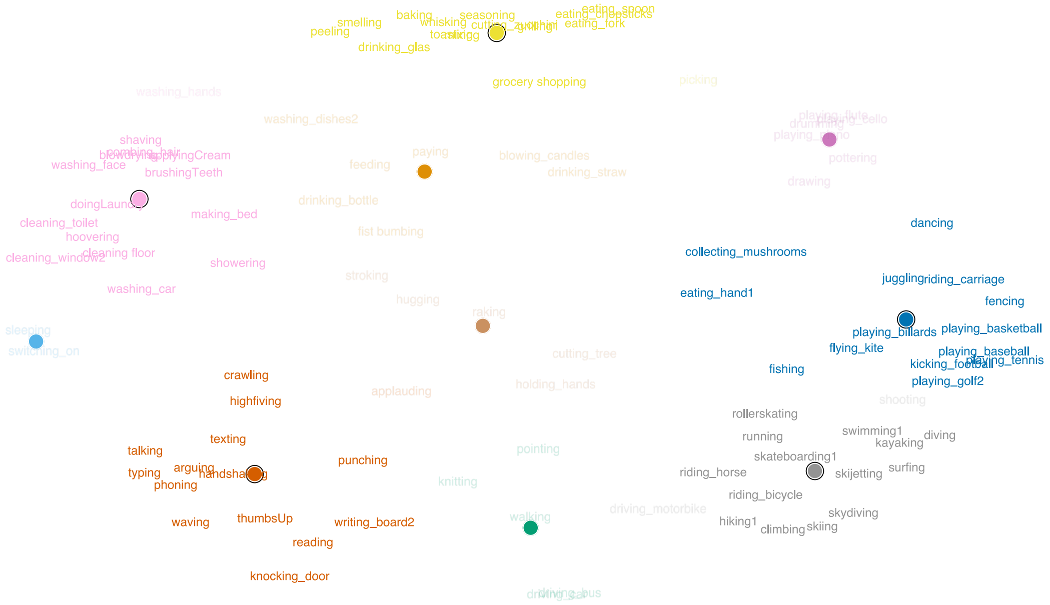

Figure S4. *Action clusters within the stimulus set obtained from the multi-arrangement task. Each word corresponds to one action image. Low distances between actions indicate a high similarity. Different colors represent different action categories, and circles show the centroid of each action category. Bright colors represent the stimuli which survived stimulus pruning. Five clusters were removed because they contained less than 10 actions. All clusters were pruned down to the size of the smallest surviving cluster by removing the actions furthest away from the centroid of the given cluster. The final stimulus set consisted of five clusters, with 14 basic-level actions per cluster. These are highlighted by black circles around the centroid.*

Interim Discussion

This experiment served the selection of an equal number of stimuli from different semantically meaningful action categories. Note that the clusters were chosen on a combination of two criteria, namely, the silhouette index and an inclusion of at least 10 stimuli per cluster. The five clusters obtained in this experiment are in line with those reported by Tucciarelli et al. (2019) and Kabulska & Lingnau (2022). Note

that these clusters were expected to be obtained, given that we chose the stimuli based on the study by Tucciarelli et al. (2019).

References

Chao, Y. W., Wang, Z., He, Y., Wang, J., & Deng, J. (2015). HICO: A benchmark for recognizing human-object interactions in images. *Proceedings of the IEEE International Conference on Computer Vision*, *2015 Inter*, 1017–1025. https://doi.org/10.1109/ICCV.2015.122

Kabulska, Z., & Lingnau, A. (2022). The cognitive structure underlying the organization of observed actions. *Behavior Research Methods*, *1*, 3. https://doi.org/10.3758/s13428-022-01894-5

Kriegeskorte, N., & Mur, M. (2012). Inverse MDS: Inferring Dissimilarity Structure from Multiple Item Arrangements. *Frontiers in Psychology*, *3*. https://doi.org/10.3389/fpsyg.2012.00245

Ma, S., Bargal, S. A., Zhang, J., Sigal, L., & Sclaroff, S. (2015). *Do Less and Achieve More: Training CNNs for Action Recognition Utilizing Action Images from the Web* (arXiv:1512.07155). arXiv. http://arxiv.org/abs/1512.07155

Monfort, M., Andonian, A., Zhou, B., Ramakrishnan, K., Bargal, S. A., Yan, T., Brown, L., Fan, Q., Gutfreund, D., Vondrick, C., & Oliva, A. (2020). Moments in Time Dataset: One Million Videos for Event Understanding. *IEEE Transactions on Pattern Analysis and Machine Intelligence*, *42*(2), 502–508. https://doi.org/10.1109/TPAMI.2019.2901464

Pedregosa, F., Varoquaux, G., Gramfort, A., Michel, V., Thirion, B., Grisel, O., Blondel, M., Prettenhofer, P., Weiss, R., Dubourg, V., Vanderplas, J., Passos, A., & Cournapeau, D. (2011). Scikit-learn: Machine Learning in Python. *Journal of Machine Learning Research*, *12*, 2825–2830.

Rousseeuw, P. J. (1987). Silhouettes: A graphical aid to the interpretation and validation of cluster analysis. *Journal of Computational and Applied Mathematics*, *20*, 53–65. https://doi.org/10.1016/0377-0427(87)90125-7

Tucciarelli, R., Wurm, M., Baccolo, E., & Lingnau, A. (2019). The representational space of observed actions. *eLife*, *8*, 1–24. https://doi.org/10.7554/eLife.47686
