# Supplementary material for: Actions at a glance: The time course of action, object, and scene recognition in a free recall paradigm
Source: Cogn Affect Behav Neurosci. 2025 Feb 26;25(3):693–707. doi: 10.3758/s13415-025-01272-6 (PMC12130074; doi:10.3758/s13415-025-01272-6)
Supplement: Supplementary file 4 — Supplementary file4 (PDF 775 KB) [file 13415_2025_1272_MOESM4_ESM.pdf]

Supplementary Material 4

Instructions: Stage One

Instructions „Images at a Glance" (translated from German)

Thank you for taking part in the experiment „Images at a Glance". In this experiment, we would like to investigate how much can be recognized on an image which is only presented very shortly. Your task will be to provide written descriptions of images which are shown to you very briefly.
Every trial starts with a fixation cross on which you must concentrate. Afterwards, you will briefly see the image which you will have to describe, followed by a strongly pixelated image without content.

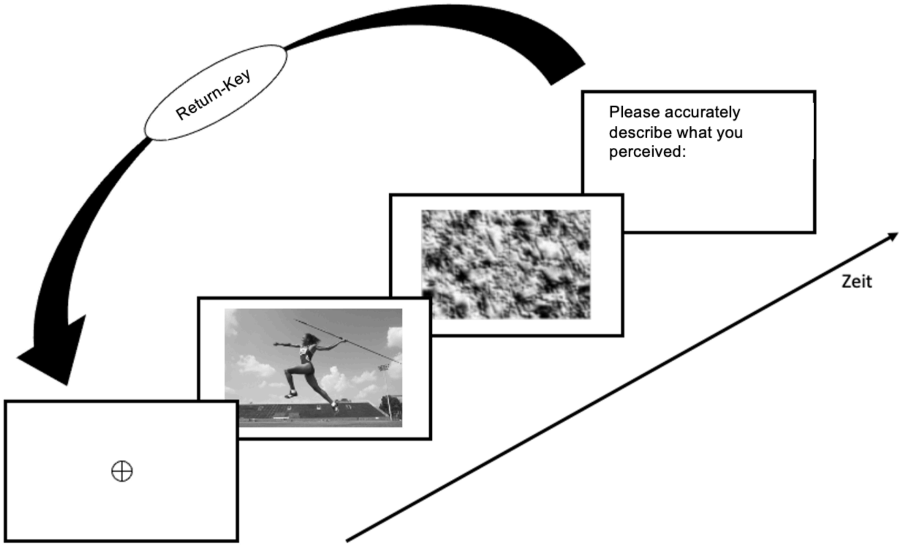

Then, you will be asked to describe what you perceived as detailed as possible. As the images will be presented for varying durations, it is possible that you perceive more on some images than on others, or that you won't be able to recognize any detailed contents due to the short presentation times. Even in this case, please accurately describe what you saw. A possible description for the image in the example would be: *"I can see a female athlete, she is wearing short, tight trousers and a t-shirt, I think with a number on it. She jumps and throws something which looks like a spear. She is outside, I could see sky in the background and something dark next to the ground, maybe a grandstand."*

Please take as much time as you need to describe what you perceived. At the end, the participants who provided the most detailed descriptions will win ice-cream vouchers – paying attention is worth it! Please be careful to not press the RETURN-key during your response, as this will stop response collection.

After pressing the RETURN-Key, response collection stops, and the next trial starts.

Do you have any questions? ☺

Instructions: Stage Two

Instructions "Image Descriptions"

Thank you for participating in our study. In this study, image descriptions are to be evaluated according to specific criteria. In each trial, you will be shown an image along with a corresponding description. Your task is to assess to what extent certain aspects of the image (action, context, object, sensory information) have been described correctly, wrong, or not at all.

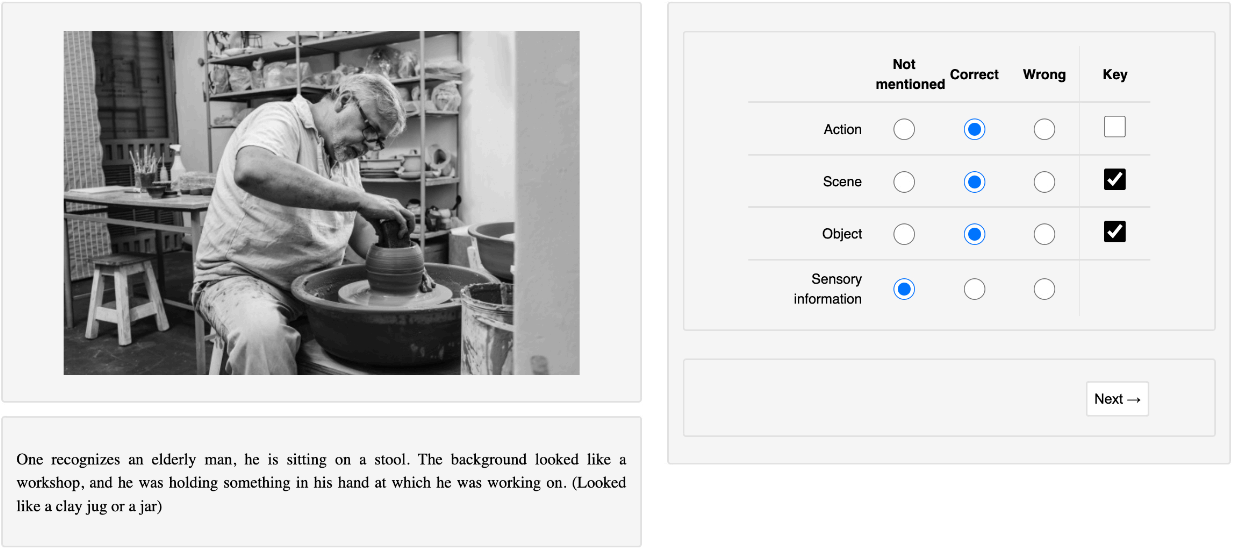

Explanation of the different image aspects:

- ***Action***: Is the depicted action mentioned, and if so, is it correctly described? Several actions may be correct. In the example shown, the following descriptions apply: the person is "*sitting*," "*doing pottery*," or "*engaging in a hobby*." Each image can also be assigned a ***central, goal-directed action***. In the current image, this would be "*doing pottery*," while in other images, it could be "*sawing*," "*cycling*," or "*baking*." Additionally, check "Key" if the central action has been correctly described.

- ***Context***: Does the text include information about the context or scene, and if so, is this information correct? In the example shown above, this would be a "*workshop*" or an "*indoor space*," while in other images, it could be a "*gym*" or a "*beach*." Most images can also be assigned to a specific category (e.g., "*workshop*," "*gym*," "*playground*"). In the example image, this would be "*workshop*." Additionally, check "Key" if the category has been correctly described.

- ***Object***: Is a <u>*non-human*</u> object mentioned, and if so, is it correctly described? In the example image, "*clay jug*," "*stool*," "*t-shirt*," "*shelf*," or "*spray bottle*" would apply; in other images, it could be a "*bicycle*" or a "*hammer*." If multiple objects are mentioned, assess whether the objects are mostly described correctly. Most images also contain a ***central object*** involved in the action. In the example image, this would be "*clay jug*" or "*pottery wheel*." Additionally, check "Key" if the central object has been correctly described.

- ***Sensory Information***: Are any sensory impressions such as shapes, textures, or colors mentioned (e.g., "*light background*," "*something dark on the right side of the image*"), and if so, do these sensory impressions match the actual image?

Once you have evaluated all criteria, press the "Next" button, and the next trial will begin. At the end of each session, you will be asked to download a file. Please download it and send it to maximilian.reger@ur.de.
