# Supplementary material for: Actions at a glance: The time course of action, object, and scene recognition in a free recall paradigm
Source: Cogn Affect Behav Neurosci. 2025 Feb 26;25(3):693–707. doi: 10.3758/s13415-025-01272-6 (PMC12130074; doi:10.3758/s13415-025-01272-6)
Supplement: Supplementary file 5 — Supplementary file5 (PDF 135 KB) [file 13415_2025_1272_MOESM5_ESM.pdf]

Supplementary Material 5

Correlations between the recognition of specific and unspecific features

Across exposure durations we did not find significant correlations between description accuracies of actions and scenes (**Figure 6-7**). To determine whether this result also holds when focusing on specific actions taking place in very specific scenes, e.g. "someone making a jar in a pottery workshop", we exploratorily computed pairwise correlations between the normalized accuracy scores of key actions and key scenes for each PT across images (see ***Correlation Analysis*** in the Methods section). For completion, we additionally computed pairwise correlations for all key-non key feature combinations at each PT. **Figure S5** shows the resulting correlations.

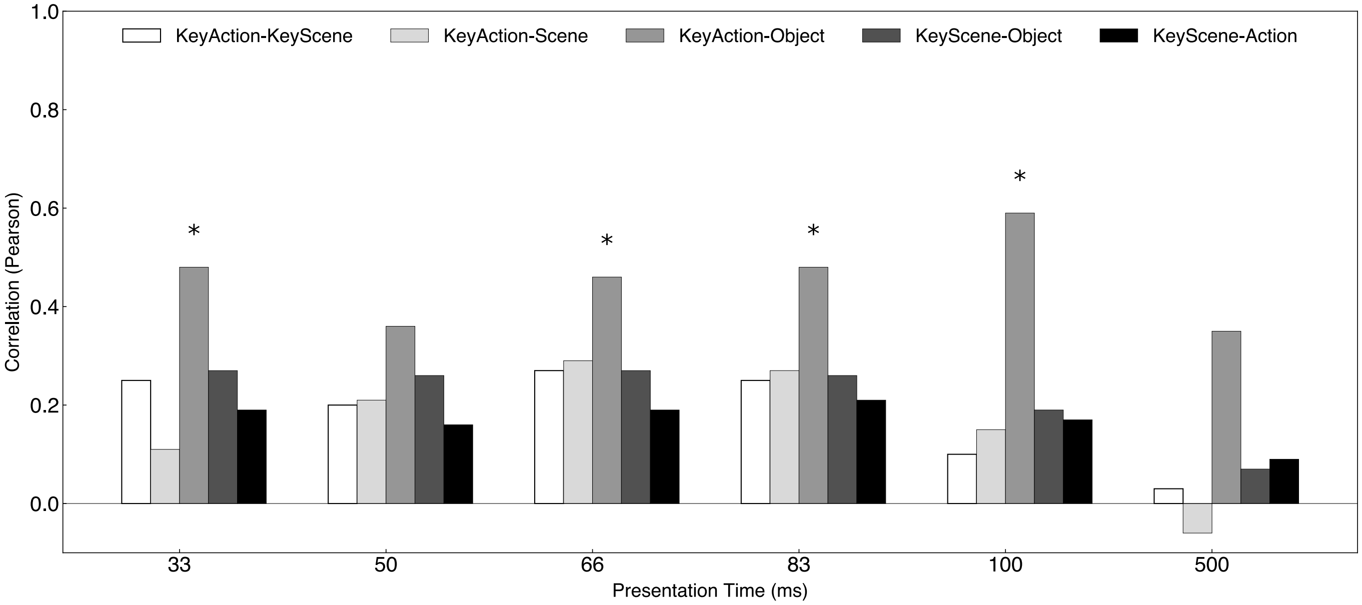

Figure S5. *Pearson correlations of normalized accuracy scores between specific and unspecific features across images, separately for each presentation time. Asterisks indicate significant differences from zero after Bonferroni correction for multiple comparisons ($p < .05$).*

In line with the results of the correlation analysis between unspecific actions and scenes, accuracy scores for key actions were not significantly correlated with those of key scenes irrespective of presentation time. Furthermore, the accuracy for describing specific

actions was highly correlated with that for the unspecific objects, resembling the pattern we saw for unspecific actions and unspecific scenes (**Figure 7**). All other specific-unspecific feature pairs did not show any significant correlations.
