# Supplementary material for: Actions at a glance: The time course of action, object, and scene recognition in a free recall paradigm
Source: Cogn Affect Behav Neurosci. 2025 Feb 26;25(3):693–707. doi: 10.3758/s13415-025-01272-6 (PMC12130074; doi:10.3758/s13415-025-01272-6)
Supplement: Supplementary file 6 — Supplementary file6 (PDF 749 KB) [file 13415_2025_1272_MOESM6_ESM.pdf]

Supplementary Material 6

Effects of low– and mid–level features on free–recall descriptions

To have an as much naturalistic stimulus set as possible, we selected images that depict actions from various perspectives and with various amounts of clutter in the background. This, however, might have brought variables that cannot fully be accounted for in our analyses. Similarly to Fei-Fei et al. (2007) we tried to account for these discrepancies by normalizing the accuracy scores with respect to the highest score achieved within each image. To test how this normalization procedure affected perceptual threshold estimation, we performed the psychometric function estimations before and after normalizing the accuracy scores. In **Figures S6** and **S7** we show psychometric functions with normalized and non-normalized accuracy scores side-by-side for *Actions*, *Objects* and *Scenes*, and for action categories respectively. As can be seen, the differences in the perceptual thresholds before and after normalization effectively do not differ in either case, with an expected shift to longer presentation times for non-normalized scores.

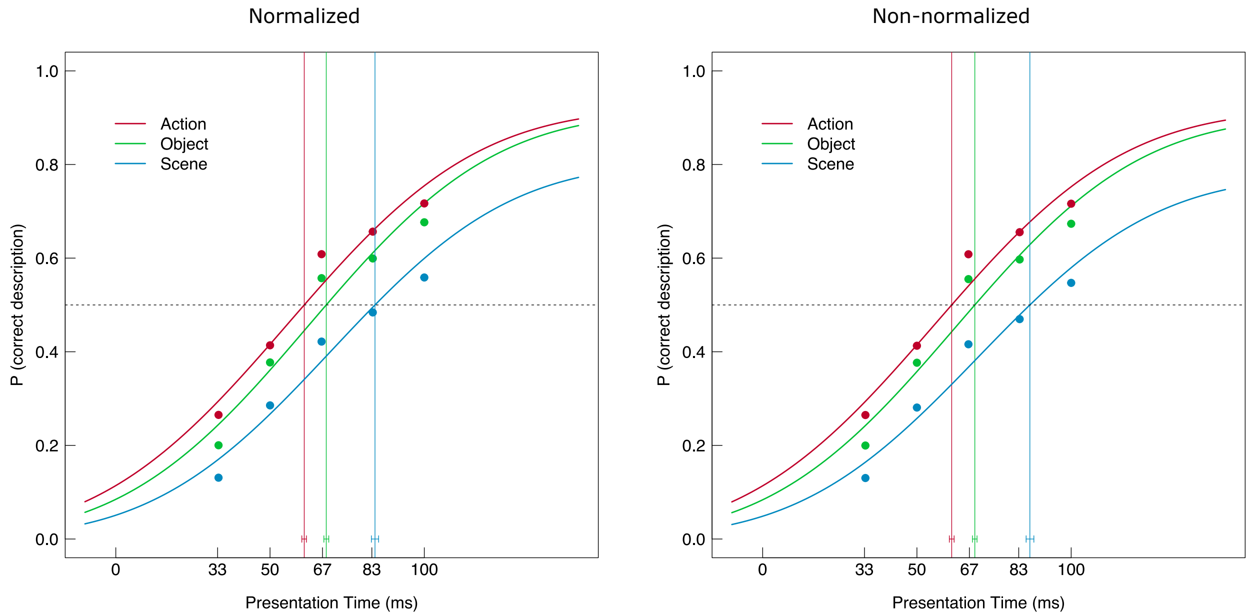

Figure S6. *Psychometric functions for actions, objects and scenes for normalized (left) and non-normalized (right) accuracy scores. Probability of a correct description of the action (red), object (green) and scene (blue) at each presentation time. Points were generated by averaging the proportion of correct feature descriptions across images and raters within presentation times. Vertical lines show the 50% thresholds. Error bars indicate 95% Cis for the 50% thresholds.*

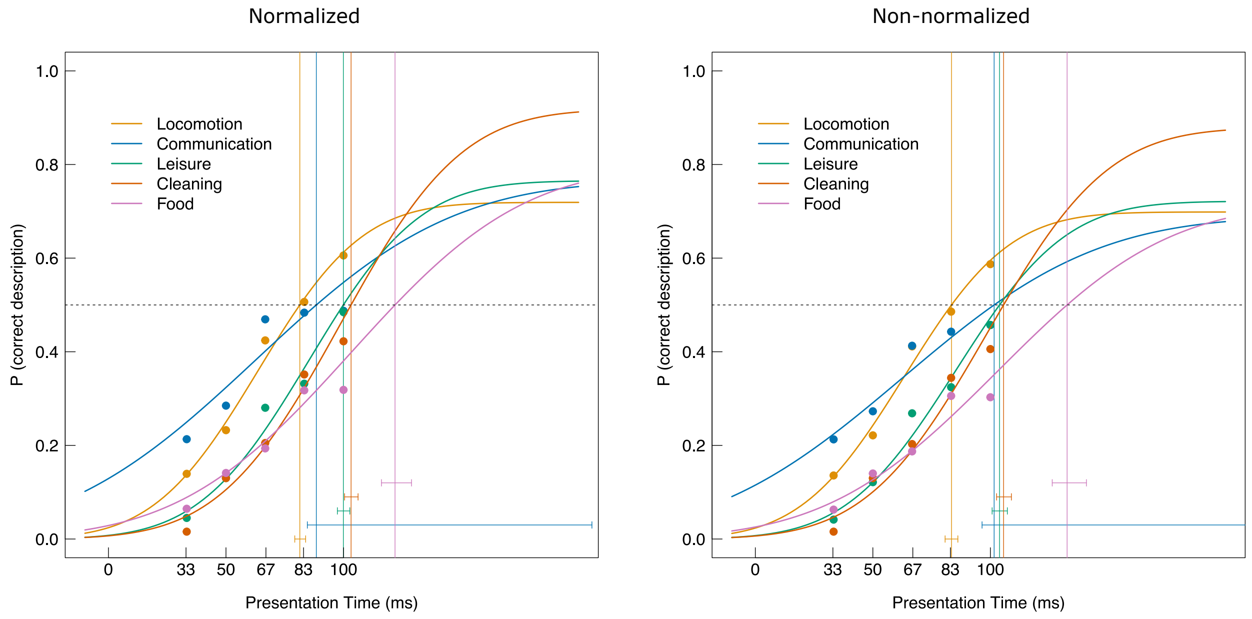

Figure S7. *Psychometric functions for normalized (left) and non-normalized (right) accuracy scores for different action categories (key actions). Probability of a correct action description for the feature "KeyAction" at each presentation time, separately for each action category ("Locomotion", "Communication", "Leisure", "Cleaning", and "Food"). Points were generated by averaging the proportion of correct feature descriptions across images and raters within presentation times. Vertical lines show the 50% thresholds. Error bars indicate 95% Cis for the 50% thresholds.*

As we showed in the regression analysis presented in **Supplementary Material 2**, stimulus complexity negatively influenced the description accuracy scores for actions, thus arguing for normalizing accuracy scores within image, to account for across image variability. However, as we showed above, the normalization procedure did not have any significant influence on the estimation of perceptual thresholds, suggesting that this

procedure might have not accounted for low-level stimulus differences as was initially expected.

To quantify the influence of low- to mid-level properties like size and position in the image, we gathered additional estimates of the size and location of the main action components (bodies, hands and objects involved in the action) in each image and then used them as predictors in a regression analysis to predict the accuracy scores for actions, objects and scenes. For each of these components we measured widths and heights (in pixels) to estimate their overall area. The positions of bodies, hands and objects involved in the action were defined at their approximate center of mass and were then converted to distance from the image center (see distributions of distance to center and relative sizes of bodies, hands and key objects in our images in **Figures S8** and **S9**). All three items have large variability in terms of their position in the images. Bodies varied in size, whereas both objects involved in the action and hands manipulating them were typically small.

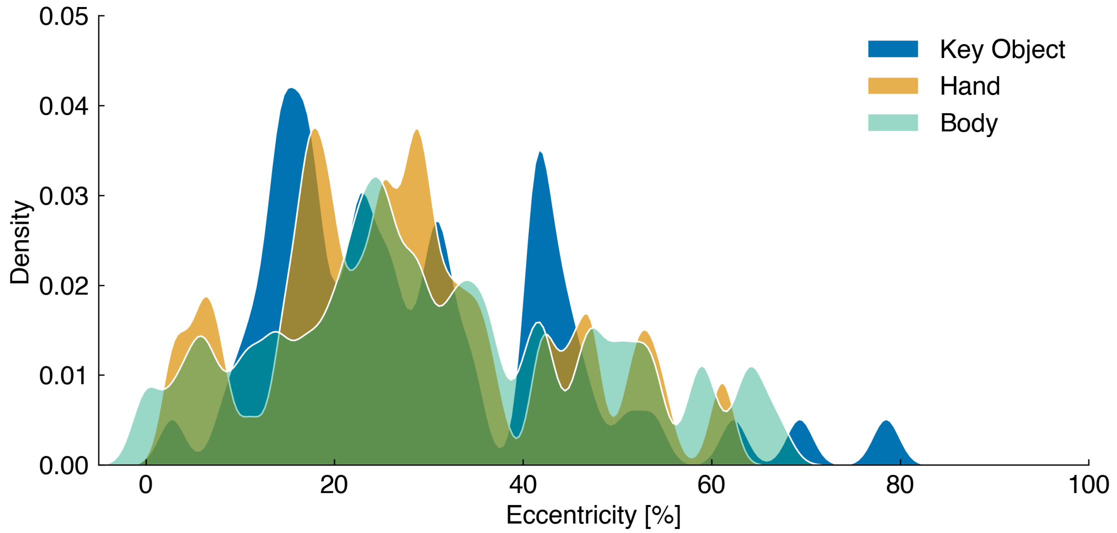

Figure S8. *Eccentricity (position relative to image center) distributions of key objects (blue), hands (yellow) and bodies (green) in our stimulus set.*

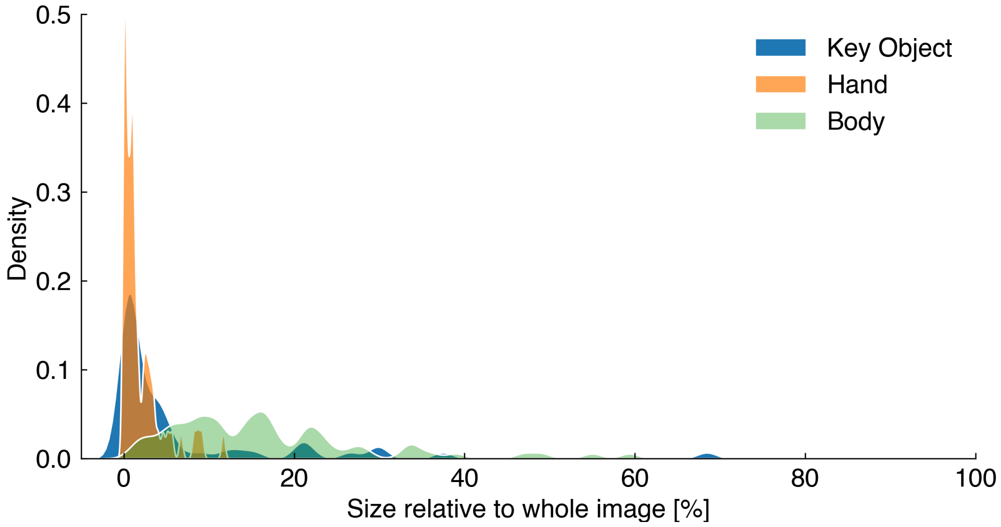

Figure S9. *Distributions of sizes of the key object (blue), hand (yellow) and bodies (green) relative to whole image in our stimulus set. Sizes were defined as the height × width of each item. Note that both hands annotated, and their sizes were summed together. In images involving two actors, sizes of their hands were summed together.*

A linear regression was then fit separately within each feature (Action, Object and Scene) with accuracy scores for each image averaged across raters as the dependent variable. Normalized accuracy scores were used here for consistency across our analyses. Sizes and distances for bodies, hands, and key objects, as well as presentation times were used as independent variables. Since not all images in the stimulus set contained all the three items (e.g. some communication images contained no key object), only images for which all estimates of size and position were acquired were used in the analysis (54/70 images). These regressors explained 50 %, 47 % and 49 % of the variance in accuracy scores for actions, objects and scenes respectively (Actions: $R^2_{adj} = 0.50$, $F(7, 316) = 45.50$, $p < 0.001$; Objects: $R^2_{adj} = 0.47$, $F(7, 316) = 40.59$, $p < 0.001$; Scenes: $R^2_{adj} = 0.49$, $F(7, 316) = 42.67$,

$p < 0.001$). In **Figure S10** we show estimated betas for each feature-predictor pair. As expected, presentation time ranked the highest, irrespective of the feature at test. On the other hand, eccentricity and relative size of bodies, hands and objects were estimated to have a relatively small negative influence on the accuracy scores. Recognition of actions was estimated to be negatively affected by object eccentricity: $\beta^{Action}_{Object\ eccentricity} = -0.23$ ($t = -3.27$, $p < .05$)) and size: $\beta^{Action}_{Object\ size} = -0.23$ ($t = -3.19$, $p < .05$). Body size negatively affected recognition of objects: $\beta^{Object}_{Body\ size} = -0.23$ ($t = -2.61$, $p < .05$) and scenes: $\beta^{Scene}_{Body\ size} = -0.32$ ($t = -3.90$, $p < .001$)).

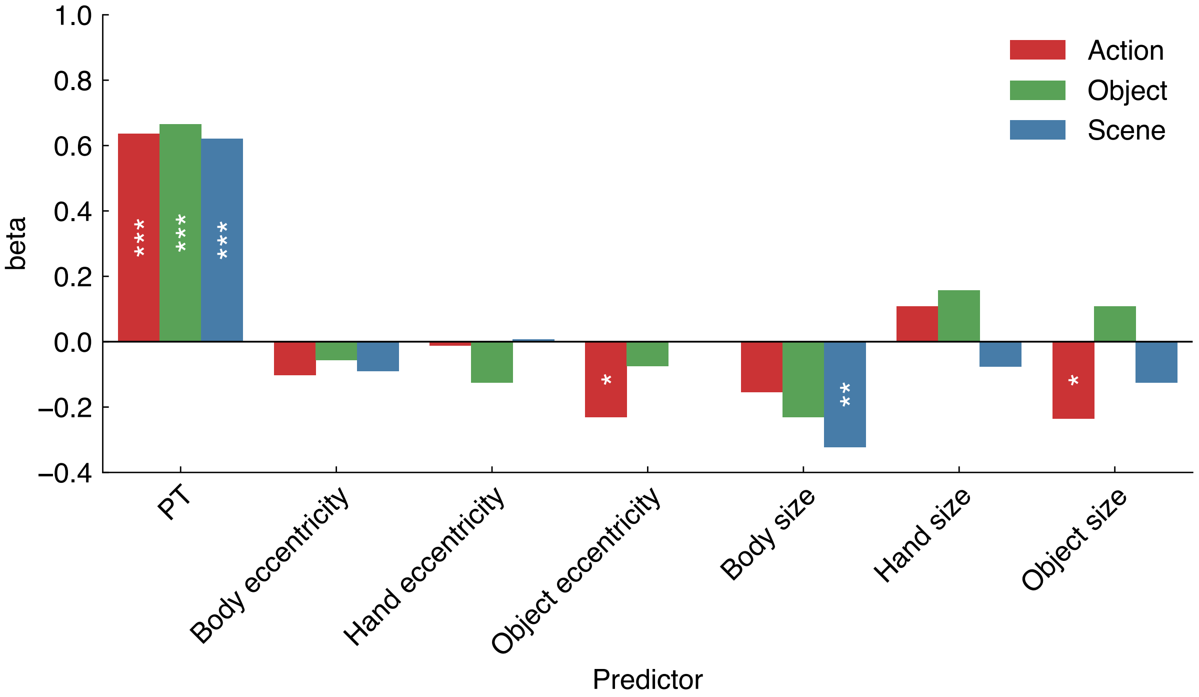

Figure S10. *Estimated regression coefficients for explaining accuracy scores of Actions (red), Objects (green) and Scenes (blue).*

To check whether accuracies for actions, objects and scenes were differentially affected by the variables depicted by these regressors, we tested whether these coefficients differed significantly between pairs of features. Thus, for each feature pair (e.g. Action - Object) we ran permutation tests in which for random samples of images we swapped accuracy scores between features as a dependent variable, fitted regressions for each feature and collected differences in the coefficients to construct a null distribution. The actual difference in coefficients was considered significant if it was smaller than the 2.5 % quantile or larger than the 97.5 % quantile of the respective simulated null distribution. Across all features pairs and regressors we did not find any significant departure from the null distribution. These results argue against the differences in size and position of action components having a strong differential effect for action-, object and scene recognition, at least in our stimulus set.

Due to the very small amount observations in addition to the imbalance in the number of images depicting objects or hands per category, we did not perform a regression within categories.
